# Abstracts of papers presented at the ISLAR (International Symposium on Laboratory Automation and Robotics) 2001

**DOI:** 10.1155/S1463924602000123

**Published:** 2002


Journal of Automated Methods & Management in Chemistry
Vol. 24, No. 3 (May–June 2002) pp. 61–98

Taylor & Francis
Taylor & Francis Group

### Abstracts of papers presented at the ISLAR (International Symposium on Laboratory Automation and Robotics) 2001

The 19th International Symposium on Laboratory Automation and Robotics provided presentations on state-of-the-art developments in laboratory automation and robotics. The symposium programme included papers and posters on all aspects of the technology. These comprised: managing laboratory automation (drug discovery); bioanalytical analysis; managing laboratory automation in drug discovery development and QC laboratory; functional genomics strategies and high throughput screening; advanced integration strategies; method development and global methods transfer; compound handling and logistics; combinatorial chemistry and automated synthesis; high throughput LC-MS-MS; increasing efficiency in dissolution testing; lead optimization; strategies for UHTS; increasing throughput for ADME toxicology; data management/data handling and bioinformatics; using contract laboratories to increase productivity; assay miniaturization; process optimization; compliance and automation—the regulatory perspective; novel high throughput screening technologies; compliance and automation—the industry perspective. Several discussion sessions were included and activated, and provided interactive communication on a wider range of subjects.

Although the programme was very comprehensive, the Symposium was designed to provide time for both formal and informal exchange of information. The technical presentations were organized into concurrent sessions with grouped papers on related topics.

Abstracts for each paper and each poster are included here. Full presentations of several of these papers will appear in later editions of this journal.

### Designing custom automation systems to fit the needs of early development

*Adam M. Fermier, The R. W. Johnson Pharmaceutical Research Institute, Raritan, NJ, USA*
*Co-authors: John Troisi, Ramon L. Rodriguez, and James V. Weber, Michael P. Graham and Erin C. Herita*

The pharmaceutical industry is under extreme pressure to reduce the costs of bringing drugs to market. Thus, to achieve this goal the entire drug development process requires continued fine tuning and the introduction of new technologies. Drug discovery has been moving through this change over the past decade. Drug development is now looking at that same daunting task of increasing throughput while maintaining compliance and headcount restraints. Custom automation systems provides one opportunity to attain this goal.

We have been investigating and building custom automation into our drug development process. For example, we have modified a Zymark Benchmate for weighing small quantities (1–50 mg) of dry powders. The addition of weighing dry powders from a stock tube enables many advantages and increases the flexibility of the Benchmate. One application we have been using this robot for is to weigh individual vials of drug substance into HPLC vials for subsequent accelerated solid state degradation studies. This typically requires about 20–40 weighings for a single study. A typical degradation program would have a dozen studies for a single compound. The weighing robot not only saves time but also offers some intrinsic safety because the analyst is not directly exposed to the material.

We have also designed a custom automated system to conduct accelerated degradation studies. These studies require precise control of sampling and temperature. A typical degradation study is usually conducted over a period of weeks. To compare studies in a relative manner for excipient compatibility studies, these times and temperatures must be maintained. It is an ideal application for automation because of its repetitive nature. The system allows for 16 reactions to be conducted in parallel. The software keeps track of temperature and times of sampling.

In this presentation we will review criteria we have found to be successful in designing the instrumentation and identifying processes that would be ideal to automate.

### Bringing Caco-2 studies from moderate- to high-throughput screening

*Dawn Alderman, Neurogen Corporation, Branford, CT, USA*

High throughput screening (HTS) technologies have been widely used in many areas of drug discovery. Human Caco-2 cells provide an *in vitro* model of drug bioavailability. They are derived from a colorectal adenocarcinoma cell line and exhibit functional characteristics of the lower small intestinal tract. They differentiate as a monolayer with apical and basal directionality that mimics the intestinal barrier. Involvement of P-glycoprotein and other active transport systems expressed endogenously influence drug absorption.

Currently, most Caco-2 studies have low to moderate throughput due to assay complexity. Many different assay formats exist and are commercially available. Becton Dickinson provides a 3-day assay system and a 10-day assay protocol. The 3-day system includes a media kit, vital to the growth and differentiation of the cells used within a 3-day period. The 10-day protocol suggests that the cells can be seeded and used after a 10-day growth period without the requirement of the enhanced media necessary for the 3-day system. The 10-day assay media that we currently use has an antibiotic in it to protect against the potential bacterial contamination that may occur with the cells seeded in the plate for 10 days. The antibiotic condition may affect the data when comparing with the 3-day system. Neurogen is currently evaluating these assays for any potential differences.

The Caco-2 model does not take into consideration first-pass metabolism. The detection method also creates a significant bottleneck. Currently LC/MS/MS is being used. By using HTS technologies, such as laboratory automation and miniaturization, we have shown that the throughput of the Caco-2 assay can be significantly increased. The introduction of the TECAN GENESIS has allowed the automation of the drug addition to the Caco-2 assay plates. It has also allowed for automated sampling. The TECAN GENESIS that is currently in our facility has 1 ml Teflon-coated syringes, and a 21-plate deck design. The programs being used have been written in-house using the GENESIS software.

We have evaluated the 3-day assay system and are currently evaluating the 10-day protocol, in 24- and 96-well formats. We have found that our data are of good quality and are reproducible in-house. We are still evaluating many aspects of the 96-well assay format. Effective cell density and the volumes used in each assay format have been assessed. We began by investigating a couple of cell densities. We have evaluated 12.5 K/well (Becton Dickinson protocol) and 25 K/well. Well volumes have also been assessed. Initially 100 μl in the apical ports and 250 μl in the basal ports was used. However, this condition is associated with a suspected wicking problem. The sample under the plate lid as well as the volume moving up the interior wall of the basal compartments has been observed. This is a possible suspect for interwell contamination. We are further developing this assay and will evaluate this phenomenon. We have attempted to cut the volumes from 100 μl in the apical compartment to 80 μl. The basal well we have cut the volume from 250 to 200 μl.

There are many assay conditions that have been published that vary between laboratories. Many publications suggest that a pH gradient from 6.5 (apical compartments) to 7.4 (basal compartments) is the best model, representing the conditions of the lower small intestine. Another assay condition that varies between laboratories is what concentration of drug to use. High concentrations of drug create the risk of saturating the P-glycoprotein transporter as well as the other active transport mechanisms. We are currently evaluating our Caco-2 studies in modified HBSS buffer at 5 μm drug concentration using the pH gradient. Again, cell density is a factor that varies between laboratories. Becton Dickinson recommends a cell density in a 24-well plate to be 200 K/well and in a 96-well plate to be 12.5 K/well. We are currently using this recommendation for both assay formats discussed.

Currently, samples are evaluated for $P_{app}$ (apparent permeability) using this published equation:

$$P_{app}(10^{-6} cm\, s^{-1}) = (V_d/A)(dC/dT).$$

This is a rate distance measurement, where $V_d$ is the volume of the donor compartment. $A$ is the surface area of the monolayer, d$C$ is the change in concentration and d$T$ is the change in time.

The Caco-2 cell model mimics the dynamics of the human lower small intestinal tract. The potential for this model to move towards a high-throughput format offers the high-throughput compound screening in the future, which is essential to modern pharmaceutical development. Optimizing the current cell density and volume specifications serve to strengthen this assay format, and is currently a major hindrance in its development.

### Rational approach for selecting extraction parameters for homogenizer-based tablet assay methods

*Alger Salt, GlaxoSmithKline, Research Triangle Park, NC, USA*

This poster will present a rational approach for choosing extraction parameters, specifically homogenizer times and speeds for tablet test methods that employ the TPW II workstation or similar homogenization-based sample extraction procedures. This approach is based on the assumption that extractions can be modelled using a first-order rate equation. The extraction profile, a curve showing the amount of drug dissolved versus time, can be characterized by a single number $k$, the first-order rate constant.

The amount of drug extracted at a given time can be estimated or predicted if the rate constant is known. Conversely, the amount of time required to reach a given amount of drug dissolved can be estimated. These assumptions allow one to model the process and predict a combination of parameters to ensure that the tablet is completely dispersed and that the analyte is extracted quantitatively. A formal protocol can then be designed and performed to validate the extraction process. The poster will describe this approach and illustrate it with a case study.

### Introduction of new technology into the pharmaceutical quality-control environment: 'cutting costs without cutting corners'

*Andy Boughey, AstraZeneca, UK*

Ever increasing demands at the drug-discovery level in recent years have lead to significant advances in automation. This in turn has lead to increasing numbers of drug candidates and correspondingly more new products being manufactured, with a subsequent impact on the Quality Assurance Department.

Automation plays a key role in the future of quality control. It is recognized that significant cost savings can be made with the introduction of automation. However, there are equally important benefits to be gained with respect to cGMP.

This presentation will discuss the pressures being placed on quality control and the benefits and concerns of introducing new technologies.

It is easy to support the introduction of new technology when the benefits can be expressed in terms of dollars saved, but what is the true value of improved regulatory compliance?

### High-throughput analysis of free amino acids in biological matrices

*Arthur Rugg, Cereon Genomics LLC, Cambridge, MA, USA*
*Co-authors: Shaoxia Yu and Lily Li*

Traditionally, chromogenic or fluorescent derivatization (FD) coupled with high-pressure chromatography (usually 20–30 min/separation) is used for amino acid analysis due the amino acids' lack of UV absorbance. To detect an alternated change in the levels of free amino acids through the screening of thousands of biological samples, the analysis of 20 amino acids using an HPLC/FD approach is tedious and time consuming. Therefore, a high-speed 1.5 min per sample LC/MS/MS method has been developed, validated and implemented to analyse 20 free amino acids—in biological matrices.

### Multitasking, scalability and flexibility: the Staccato™ automated workstation

*Matt Boeckeler, Neurogen Corporation, Branford, CT, USA*

With drug-discovery technology advancing and changing as rapidly as it is, there is a growing need for automated equipment to have the capability to change with these changes in technology. The use of single-workstation approaches to liquid handling is decreasing due to the increasing need for a system that can perform various liquid-handling tasks on the same platform.

The Staccato™ automated workstation is an extremely versatile liquid handler with the capabilities of handling plate formats ranging from 96- to 1536-well plates. The 'Drag and Drop' architecture of the software allows for flexibility and ease in process development. With the use of CLARA™ 2001 software, method development and system reconfiguration become less intensive. Its scalability lends itself to supporting changes in platforms as emerging technologies arise.

The Staccato™ system can be equipped or configured with many components and options to fit your liquid-handling needs. Using the SciClone ALH as the base liquid handler, the system can come equipped with a disposable tip Rapid head, or a small volume fixed tip head with 96 or 384 cannula arrays. To augment the liquid-handling capabilities of the SciClone, up to four separate Cavro™ bulk-dispensing manifolds can be mounted on the head for reagent additions. Liquid handling can be further enhanced by the addition of an eight tip, independent *Z*-axis probe used for various liquid-handling applications. Integration of the Autostack with the SciClone gives an exceptionally large storage capacity for unattended operation. The use of on-board barcode readers in conjunction with the CLARA™ Data Manager, gives the system the ability to track all compound and assay plates entering the system.

Reformatting chemical libraries with the Staccato™ system is very efficient. With its large storage capacity and flexible configurations, reformatting can be done through a variety of methods. Using the Rapid head configuration, 300 96-well plates can be reformatted to 75 384-well plates in one 8-h day. Using the small volume fixed-tip configuration, one can convert 200 96-well plates to 50 384-well plates in one 8-h day. Although fixed tip configurations yield less throughput due to implementation of wash protocols, there are no consumable costs. It also gives you the ability to reformat further from 384- to 1536-well plates, with an even smaller volume 384-cannula array designed for 1536 applications.

The independent *Z*-axis probes provide the ability to cherry pick select compounds, assemble serial dilution plates, and manipulate compounds and reagents for ADMET studies. Performing parallel processing of plate types using the 'on the fly' array swap capabilities, the Staccato™ is capable of reformatting from 96- to 384-well plates using the 96-cannula array. After dropping off the 96 array, it can then pick up the 384 array and further dilute or deliver compounds directly to assay plates for execution with no human intervention.

The presented pipetting specs are based on five trays run with three transfers performed per tray. A 0.5 µl wet transfer produces excellent precision with an average %CV of 6.4, and an accuracy range of 1.06–7.3%. A 0.5-µl dry transfer yields an average precision %CV of 8.6, and an accuracy range of 0.03–10.1%. Dry transfers of 1 µl yield a precision average %CV of 5.8 and an accuracy range of –1.5 to 4.4%. Volumes > 1 µl yield similar results to the 1-µl numbers.

System use at Neurogen Corporation entails taking in chemical entities from our existing archive or from our high-speed synthesis group and performing compound dilution procedures, or reformatting processes on the plates and transferring the compounds directly into assay plates, all within the same method. On this platform, the system can also build dose–response plates, prepare compounds for interdepartmental support or execute a variety of assays.

After compounds are in the proper format and concentration, with the use of the Cavro™ dispensers, multiple reagent addition to assay plates can be accomplished with the proper scheduling of processes. In essence, the system can take compounds from their starting format and concentrations, manipulate the plates to accommodate assay-specific plate formats and conditions, then proceed to add the appropriate reagents via the Cavro™ dispensers, bringing the assay from compound delivery, through reagent addition. Dispensing by the Cavro™ syringes for volumes of 10–2500 µl all yield similar results. Across plate precision yields an average%CV of 3.8 with an accuracy range of 1.1–5.5%. Non-contact dispensing of reagents eliminates cross-well contamination and with multiple manifolds, various reagents can be used.

Replication of compound data generated by the 384-cannula array at a dry transfer volume of 0.5 µl is quite good. Dry dispensing of such a small volume is not easily achieved, but obtainable with acceptable %CV using particular pipetting methods. Using the same pipetting methods and comparing data to proven control methods for drug delivery yields very acceptable %CVs and correlation.

CLARA™ 2001 Software is used for scheduling methods and integrating peripherals into the existing system. It communicates with adapter modules that translate commands between CLARA™ and the ICP of the peripheral module, triggering program execution of the instrument. This method of integration makes the scalability of the system much easier and more efficient than previous conventional methods. Upon process completion, the ICP sends a message back to CLARA™ through the adapter to confirm completion, or send notice of an error in execution. The system's open architecture, in conjunction with the CLARA™ software, allows for flexibility and ease when modifying or implementing new assay platforms and technologies.

The CLARA™ Data Manager gives you the ability to manipulate and filter incoming data with the use of the Data Manager's ZyMap. Data can be configured into text files, Excel sheets or directly transported to a database via ODBC connections. However, the current version of the Data Manager is unable to catch critical events—events that would trigger the data to be sent to the database, such as an 'end of run' event. This inability does not yet allow the direct triggers to export data to a database. A temporary remedy supplied by Zymark is a developer's kit (a collection of APIs) that gives you the ability to build your own Data Manager using predefined templates. Using this method could require extensive time and programming on the user's end depending on the process and data being captured. However, an updated version of the Data Manager is anticipated to be released at the end of October that would alleviate this problem.

Although the Staccato™ system has many benefits and capabilities for liquid-handling needs, there were some implementation issues along the way. The biggest setback experienced was with limitations of the Data Manager. One of the top reasons for the selection of the Zymark system over other vendors' was the ease of database connectivity and data export. Another issue was in the design of the fixed tip arrays. Great care must be taken not to damage their Teflon coating. Any scarring or scraping of the material will cause variation in pipetting procedures especially at small volumes. Service for the most part is good; however, the redesign and complexity of the system left the need for more time in training of technicians to come up to speed in the overall workings of the system.

Overall, Neurogens experience with the Staccato™ system has been a good one. All systems have their benefits and drawbacks. The most important thing is the cooperation and speed of assistance received when problems do arise and efficiency in coming up with solutions. In this aspect, Zymark has shown dedication and commitment to their products and customers.

### Update from PQRI's Blend Uniformity Working Group: balancing workload with batch homogeneity assurance

*Garth Boehm, Purepac Pharmaceutical Co., Elizabeth, NJ, USA*

The Product Quality Research Institute (PQRI) is a consortium between industry, academia, and FDA, which aims to provide a scientific basis for the development of regulatory policy. One of PQRI's first initiatives was to form a Blend Uniformity Working Group (BUWG) charged with providing a scientific basis for continued development of FDA policy on BU testing. The BUWG sought wider industry input through a survey of current BU testing practices, and a public Workshop. Using this input together with the experience of the BUWG members and colleagues, a draft proposal has been developed and refined. The proposal provides a guide for appropriate testing during scale-up and validation, and for subsequent routine manufacture. The proposal is based around initially establishing the relationship between BU testing and stratified testing of the resulting dosage units. The type and amount of testing recommended during routine manufacture depends on the outcome and robustness determined from the validation testing. The BUWG is currently seeking data from industry to challenge the proposal with real data to establish the validity of the suggested approach. The next step will be to seek public comment on the BUWG proposal.

### Views from the past: HTS in the Molecular Biochemistry Department, Glaxo Wellcome, Inc.

*Brent Butler, GlaxoSmithKline, Research Triangle Park, NC, USA*
*Co-authors: Cole Harris and Steve Blanchard*

The pharmaceutical industry saw a tremendous increase in screening throughput in the 1990s with the introduction of advanced robotics and liquid-handling systems, as well as homogeneous assay formats conducive to higher density assay plates. During this time, there have been numerous debates on the advantages and disadvantages of centralized versus decentralized screening sites. There have also been discussions about the use of large, integrated robotic systems compared with small bench-top robotics.

Glaxo Wellcome Research Triangle Park has screened in a decentralized manner using both large automation platforms and small bench-top systems. In addition, the screens have migrated from 96- to 384-well plates. This talk will focus on the lessons learned from the screening effort in the Molecular Biochemistry Department of Glaxo Wellcome.

### Fully automated screening of intracellular calcium using a novel detector

*Brian Rasnow, Amgen, Inc., Thousand Oaks, CA, USA*
*Co-authors: Jack Lile, Karen Kearns, James Treanor and Peter Grandsard*

We have constructed a fully automated high-throughput, cell-based screening system that measures aequorin luminescence kinetics as an indicator of intracellular calcium concentration. The system uses a modified Zymark Rapidplate96 pipetting station to add drug candidates and known modulators to aequorin-expressing cells grown in microtitre plates. Simultaneous with compound addition, custom optics and a high-performance chilled CCD camera added to the Rapidplate96 take an image sequence of the plate bottom. Compounds that affect calcium entry or release from intracellular stores can be analysed using this system. Agonists and antagonists can be detected in the same screen by imaging both during compound addition and during a subsequent addition of known agonists.

The 96-channel imaging flash luminometre is integrated with a Beckman $CO_2$ incubator, Zymark Twister, tip store, ORCA robot, and other peripherals to perform fully automated screening of up to 72 microtitre plates with a cycle time of several minutes per plate (about 10 000 high-content, cell-based assays/system per day). The system is controlled with Amgen's automation software ('Synchronicity'). Analogue and digital signal processing are performed in real time to remove artefacts from cosmic rays and bad pixels, and normalize any systematic effects from geometrical and biological heterogeneity across the plates. This reduces the about 1.5 MB/plate of raw data to about 10 kB of time series, which can be further compressed and the compounds classified using principal component analysis and other methods.

We have validated the system for ion channel and GPCR HTS and dose–response characterization, and it has screened thousands of plates. We will discuss and contrast its design and performance with other devices that have comparable capabilities, and explore some of the data and information processing challenges presented by high-throughput, high-content screening systems.

### Automated production of medicinal agents from plant matrices

*Bruce E. Richter, Dionex Corporation, Salt Lake City, UT, USA*
*Co-author: Richard E. Carlson*

Interest in the pharmacologically active compounds found in plant tissues is growing. The extraction techniques normally used to remove these compounds from plant tissues require long periods and copious amounts of solvents. Accelerated solvent extraction (ASE) has been proven effective in removing target compounds from a variety of plant tissues. Using ASE, the extraction of compounds from medicinal plants is completed in about 15 min using only 20 ml solvent. Extraction of the target compounds may be only a part of the isolation process. Interfering compounds such as waxes, pigments and tannins can be coextracted.

These interferences must be removed before the extracts can be subjected to screening techniques. Solid-phase extraction (SPE), liquid–liquid extraction (LLE) and preparative liquid chromatography are the techniques most often used for removing interferences from plant extracts. However, these steps are separate and not coupled with the extraction process.

This presentation will discuss the coupling of ASE with liquid-handling apparatus to produce plant extracts free from interfering coextracted compounds. This combined system produces natural product extracts that are ready for biological or chromatographic assay. The comparison of results from this procedure to those obtained without automation will be discussed.

### Determination of cytotoxic effects of different compounds using a multiparametric cell-based cytotoxicity assay

*Chandrasekaran Vasudevan, Cellomics, Inc., Pittsburgh, PA, USA*
*Co-authors: Oleg Lapets, Nancy Kerkvliet, Greg LaRocca, Rachael Krally, Lisa Lemmler, Patricia Petrosko, Megan Weiss, Joseph Zock and Richik Ghosh*

*In vitro* screening of active compounds for toxic effects earlier in the drug-discovery process will help identify problems and reduce the high failure rate of current clinical candidates. Cell-based toxicity assays are crucial in this effort, but are limited by the result generated, usually cell death. Thus, we have developed a more sensitive multiparameter cell-based cytotoxicity assay that quantifies early changes to key aspects of cellular physiology that can lead to cellular toxicity.

This assay uses our ArrayScan®HCS System High Content Screening (HCS) platform that simultaneously measures changes in nuclear morphology, cell permeability and lysosomal physiology for individual cells, and also changes in cell density in microplate wells. Dose–response and time-course data demonstrating the assay's multiparametric nature will be presented for sample compounds that affect the cellular targets in various cultured cell lines and also rat primary hepatocytes. Our data show the assay's ability to capture cellular reactions to compounds and correlate multiple toxicological indicators at a single-cell level. This assay represents a distinct advantage for HCS as a screening tool early in the drug-discovery process.

### Development of a high-throughput, homogeneous, cell-based assay for screening inhibitors of multiple drug-resistance pumps on FLIPR or FLEX-Station systems

*Christopher Silva, Molecular Devices Corporation, Sunnyvale, CA, USA*
*Co-authors: Kelly J. Cassutt, Anne T. Ferguson and Jesse Twu*

Multiple drug resistance (MDR) pumps expressed on the surface of normal cells are involved in eliminating toxic compounds generated by or exogenously introduced to the cells. In cancer chemotherapy, these pumps may become over-expressed by the tumour cells and render the drug treatment ineffective following relapse. MDR pumps are also normally expressed at the blood-brain barrier (BBB) and as such give the brain 'sanctuary' from chemotherapeutic drugs.

Our goal at Molecular Devices is to provide a tool to screen libraries for compounds that inhibit the MDR pumps. Such compound leads could be important for inhibiting MDR, which might otherwise lead to failure of the therapeutic regimen. The compound leads identified might also help facilitate the absorption or retention of other useful therapeutics for disease treatment.

The assay is designed primarily for screening inhibitors against the two most common MDR pumps, P-glycoproteins (P-gps) and multidrug resistance-associated proteins (MRPs). Both classes of pumps are known to expel a variety of mechanistically and structurally unrelated cytotoxic drugs such as anthracyclines, tacanes, vinca alkaloids, and epipodophyllotoxins as well as some fluorescence substrates or indicators from the cells. For a given assay the compound library is prepared in a 96- or 384-well plate and allowed to incubate with cells at room temperature for 15 min or longer. The microplate is placed in the FLIPR or FLEXStation System followed by addition of the fluorescence indicator by the instrument. The kinetic readout of the results can then be collected in a real-time mode. The results are expressed as the change in relative fluorescence units (RFU) over time, usually 5-8 min after the start of the experiment. Inhibition of the MDR pumps is indicated by the increase in RFU above the baseline control.

Using the cell line MCF-7/ADR* (resistant to adriamycin), we showed that the baseline change in counts without inhibitor is nearly zero while 30 mM cyclosporin A (a known inhibitor to P-gp) increased the RFU to $> 5000$. These results indicate that the P-gp expression level is high enough to keep dye completely out of the cells and the MDR pumps were inhibited effectively by cyclosporin A. Various tumour and non-tumour cell lines including the parental MCF-7, Caco-2, and T-cell line CEM were also tested and showed parallel results. Very little or no DMSO interference to the assay was observed.

We have developed a robust and reproducible homogeneous cell-based assay for high-throughput screening of compound libraries for inhibitors of MDR pumps. The ease of use inherent in the format with no wash step involved along with the rich kinetic data previously unavailable to the large-scale MDR studies indicate that the FLIPR MDR Assay Kit will be a time-saving and cost-effective tool in HTS screening of inhibitors of MDR pumps.

### Custom hit-picking robotic system

*Claude Dufresne, Merck Research Laboratories, Rahway, NJ, USA*
*Co-authors: Christopher Napolitano and Keith Silverman*

An in-house integrated automated ‘cherry picking’ system will be described. It is designed to provide backup samples of natural products’ ‘hits’ to biologists performing primary screening. The system uses a Tomtec MegaStor for high-capacity source plate storage. The system is controlled by custom software, written in Visual Basic 6.0, and makes use of ActiveX controls for each of the system components.

### Direct analysis of basic drugs in cell culture lysate using online extraction LC/MS/MS

*Claude R. Mallet, Waters Corporation, Milford, MA, USA*
*Co-authors: Jeff Mazzeo and Uwe D. Neue*

During the last 10 years, pharmaceutical companies have constantly pushed for shorter analysis time to breach the 1000 analyses per day barrier. With this demand for high-speed analysis, new techniques such as 96-well plates, fast gradients or ultrahigh-flow chromatography are showing promising results.

We have focused on online extraction techniques for high-throughput analysis. In a previous study we investigated the potential of online extraction for the analysis of basic and acidic drugs in rat plasma. Recently, we have turned our attention to other types of matrices, e.g. the study of toxicity of drugs in cell cultures used in pre- and post-discovery phases. Several chemical and physical lysate methods were evaluated. The extracts were injected onto an extraction column at high flow rate (i.e. 4 ml $min^{-1}$) [1–3] to remove macromolecular compounds such as proteins, but trap smaller analytes on the head of the column. Several configurations for direct injection are possible. In the simplest configuration, the extraction column is connected directly to the MS/MS system. Other versions are configured with a single or a dual extraction column coupled to an analytical column. It is often necessary to split the flow. However, in cases where sensitivity is low, this option is not recommended. For efficient high-speed analysis, the use of a second pump and a 10-port valve is also a good choice. One line (high flow rate) can be dedicated to the extraction column, while the other (low flow rate) drives the analytical column and the mass spectrometer.

A three-valve configuration using two extraction columns was used for the analysis of basic drugs in cell culture lysate. The online analysis was performed on an Oasis HLB extraction column ($2.1 \times 30$ mm, 25 μm) using a Waters Alliance 2790$^{TM}$ in the gradient mode and a 515 stand-alone pump in the isocratic mode. The extracted analytes were forward flushed into an XTerra column ($2.1 \times 30$ mm, 3.5 μm), which was added to provide additional separation power. The drugs were quantified using a MicroMass Ultima$^{TM}$ triple-quadrupole mass spectrometer equipped with an electrospray source and set in multiple reaction-monitoring mode (MRM).

### References

1. Xia, Y. Q., Whigan, D. B., Powell, M. L. and Jemal, M., 2000, *Rapid Commun. Mass Spectrom.*, **14**, 105.
2. Ayrton, J., Dear, G. J., Leavens, W. J., Mallett, D. N. and Plumb, R. S., 2000, *J. Chromatogr. A*, **828**, 199.
3. Eeckhout, N. V., Perez, J. C., Clearboudt, J., 2000, Vandeputte, R. and Van Peteghen, C. *Rapid Commun. Mass Spectrom.*, **14**, 280.

### A look to the future: screening in the Systems Biology Department, GlaxoSmithKline, Inc.

*Cole O. Harris, GlaxoSmithKline, Research Triangle Park, NC, USA*
*Co-authors: Brent T. Butler, Steven G. Blanchard and David C. Morris*

Screening at the newly formed GlaxoSmithKline Research Triangle Park site will be heavily dependent on optimizing the successful processes incorporated over the previous 5 years in the Molecular Biochemistry Department at Glaxo Wellcome. This will require expansion of the developing approaches to research aided by successful utilization of existing automation equipment. GlaxoSmithKline will compartmentalize these processes to exploit the benefits of specific strategies, further defining the process of moving chemistry towards hit identification. This discussion will focus on accommodating a larger infrastructure that provides greater target numbers and larger libraries, as well as complete follow-up screening using the automation resources available.

### Distributing mammalian cell lines for HTS using the Hydra-96 Microdispenser

*Courtney T. Ward, MDS Pharma Services, Bothell, WA, USA*

Reliable and consistent distribution of cells into microplates is a key aspect of the performance of cell-based screening assays. Typically, this has been done using a hand-held multichannel pipettor in a biosafety cabinet, a task that is both labor-intensive and slow. We have found that this step can be accelerated significantly using a 96-channel pipettor such as the Hydra-96 Microdispenser whose physical profile permits the use of the instrument in a standard tissue-culture hood. This device consists of an array of 96 glass syringes (290 µl) with fixed needles where the plungers move in unison under microprocessor control. Using four mammalian cell lines, we compared the pipetting performance of the Hydra-96, a hand-held pipettor, and a disposable tip-based 96-channel pipetting workstation. The results indicate that the well-to-well dispense precision and cell viability are equivalent for all three devices.

### Automated offline solid-phase extraction for polar new chemical entities using the Zymark Rapid Trace™ systems

*Daksha Desai-Krieger, R. W. Johnson PRI, Springhouse, PA, USA*

A generic automated offline SPE method using the Zymark Rapid Trace™ systems was developed for three very polar new chemical entities (NCEs) undergoing development at RWJPRI, namely a neuroaminidase inhibitor, a cephalosporin and a thrombin inhibitor. The polar characteristics of these compounds made it impossible to carry out liquid–liquid extraction as a sample clean-up step for these compounds.

Our goal was to obtain simple automated SPE methods as sample clean-up for these polar compounds from biological matrices and develop quantitative LC-MS/MS assays for these compounds. Challenges encountered during assay development and details of the method will be presented. Transfer of these automated offline assays to online automated SPE and/or offline automated 96-well SPE will also be discussed.

### Development and validation of an automated pooling process for a compliant preparation of blood plasma pools destined for molecular testing

*Danuta Kierek-Jaszczuk, Cangene Corporation, Winnipeg, Manitoba, Canada*
*Co-authors: Mike Labossiere, Beverly Belanger, Patti Hosler and LeeAnne Macaulay*

Blood-borne viruses such as HCV, for example, pose a danger of being transmitted from affected donor(s) onto the recipients of the blood/plasma or thereof derived pharmaceuticals. The use of nucleic acid testing/nucleotide-amplification testing (NAT) was shown to shorten the 'window period', i.e. a period when donors are in a state of active but serologically undetectable infection. When applied to the plasma pools assembled from the donations destined for the manufacture of the plasma-based therapeutics, HCV-specific NAT would decrease the risks of HCV transmission onto the recipients of such therapeutics if virus-contaminated donation(s) are identified and removed from the fractionation pool.

Accordingly, an automated plasma-pooling process and pooling-driven back-tracing algorithm have been developed and validated at Cangene to support the production of WinRho and VariZig therapeutics bearing lesser risk for HCV transmission. The validated Microlab ATplus 2 automated liquid handler has been used for the preparation of the plasma mega (master) pools in a three-stage process where final mega pools were assembled from the intermediary secondary pools and these, in turn, from the subpools. The automated barcode-reading function of the pooling instrument, in a combination with a suitable barcode-labelling system of the individual plasma donation- and pool-holding containers, allowed for the positive identification of both the individual donations and assembled pools. The pipetting reports generated by the instrument facilitated a full traceability of all pipetting events whereas gravimetric in-process verification assured pipetting accuracy.

A pooling scheme-driven back-tracing algorithm has also been developed and validated for singling out the individual contaminated donation(s). This algorithm involved four-stage back-testing and was based on the assumption that all NAT-positive test results are true-positives. It allowed for identification of viral contamination of a single donation by testing 26 samples, i.e. one final pool, five secondary pools, 12 subpools and eight individual donations. The developed and validated processes allow for cGMP-compliant preparation of the plasma pools and regulatory required back-tracing of the viral contamination to individual plasma donation(s).

### Extending the reach of miniaturization technology across the screening laboratory

*David A. Dunn, Pharmacopeia, Inc.*
*Co-authors: James R. Beasley, Paul McCoy, Barbara Strohl, Robert Swanson, Tiffany Walker and Jingchun Yang*

Assay miniaturization and the implementation of high-density 1536-microwell screening is vital to our need to increase the efficiency of primary screening and early phase lead discovery. To serve this need, we have developed a 1536 uHTS platform that employs microwell plates, novel fluid handling and optical detection technologies. Full use of this platform requires the ability to develop assays efficiently for a variety of biological targets in a straightforward and expeditious manner. Having a portfolio of miniaturizable assay technologies that can be easily developed into ultrahigh-throughput screens for any member of a class of biological targets would enhance and extend the utility of this technology. We have evaluated a variety of assay technologies that are suitable for uHTS of kinase and GPCR targets. This presentation will discuss the relative merits of these technologies in assembling a portfolio of uHTS assays for screening these target classes.

### Prequalification of pharmaceutical leads

*David Casebier, ArQule, Inc., Woburn, MA, USA*

Reliable predictive models and increased throughput of ADME screens currently enable the design, construction and profiling of chemical libraries, providing confidence for series selection in hit-to-lead discovery. Predictive models for metabolism, absorption, BBB as well as diversity measurements assist in library design and reagent selection. Robotic syntheses then generate spatially addressed, high-quality libraries, which in turn are profiled for ADME characteristics. Integration of these technologies allows for the prioritization of lead series using data indicative of developmental survivability.

### Automation of identification and screening of genomic targets related to psychiatric disease

*David M. Evans, Psychiatric Genomics, Inc., Gaithersburg, MD, USA*

Psychiatric Genomics, Inc., is interested in discovering the underlying genetic causes of psychiatric diseases and identifying therapeutics for them. Many of these diseases are multigeneic in nature and have not previously been well characterized owing to the difficulty in obtaining the appropriate tissue samples and the lack of technologies that could detect the changes in gene expression profiles within the brain regions.

In recent years, several tools and technologies have been developed that allow multiple parameters to be tested in a single experiment. Micro-array technology allows the discrimination of changes in many genes at one time, and by comparing 'normal' versus diseased tissue, it is possible to identify gene expression patterns quickly that are altered due to the disease. Having identified the disease gene profile, it is important to be able to develop screens to examine whether small chemical compounds can revert the gene profile of the diseased state back towards 'normal'.

This presentation will describe some of the approaches being taken by Psychiatric Genomics, Inc., to automate each of the steps in the drug-discovery process using the new paradigm of automated drug discovery—'Multi-Parameter High Throughput Screening' (MPHTS$^{TM}$).

### Comparison of ELISA between automation and manual testing for measuring ARANESPTM in rat serum

*De Chen, Amgen, Inc., Thousand Oaks, CA, USA*
*Co-authors: Yan Wang, Monica Zordich, Han Gunn and Sharon Baughman*

This presentation compares the results of measuring ARANESPTM in rat serum, obtained manually or via a fully automated immunoassay system. ARANESPTM is a new erythropoietic protein currently used in clinical trials for the treatment of anaemia related to chronic renal failure.

The Tecan Genesis workstation 200 was developed to perform the ARANESPTM quantitative ELISA. It was equipped with eight pipetting ceramic needles capable of pipetting 5–1000 μl, a shaking incubator, a Columbus microplate washer, microplate hotels and a microplate reader. A robotic arm transported microplates and lids between the peripherals on the workstation deck.

Seven independent studies were performed on various days by the TECAN and manually. Standards, quality controls and known samples were loaded in triplicate on the plates in each assay. The data generated showed no significant difference. ANOVA analysis confirmed this conclusion.

Fully automated systems can be introduced to quantitative immunoassay for protein measurement in serum. Technical advances in methodologies, robotics and computerization will lead to significant enhancements in the capacity of immunoassay, resulting in significant cost and efficiency gains.

### LC-MS/MS assay for the quantitation of RWJ-270201 using the Zymark Rapid Trace SPE workstation

*Denise Preston, R. W. Johnson PRI, Spring House, PA, USA*
*Co-authors: Daksha Desai-Krieger, Virginia Scott and R. John Stubbs*

A simple, automated SPE extraction and LC-MS/MS assay was developed for the extraction and quantitation of two NCEs, RWJ-270201 and RWJ-270204, being evaluated as viral neuroaminidase inhibitors intended for use in the treatment of influenza A and B. A solid-phase extraction method was developed for these analogues using Zymark Rapid Trace SPE workstations.

Extraction of plasma samples was performed using IST Phenyl SPE cartridges (200 mg 3 ml$^{-1}$) conditioned with methanol and water. Sample clean-up was achieved by rinsing cartridges with methyl-*t*-butyl ether. The compounds of interest were eluted using ethanol:water (90:10

v/v) and subsequently evaporated under nitrogen to dryness. The residue obtained was reconstituted before injection on an LC-MS/MS in the positive-ion APCI mode.

The developed assay demonstrated good precision and accuracy and was linear over a broad curve range. The method developed for these analogues was routinely used to support preclinical pharmacokinetic studies and was later adapted for use with 96-well plate technology in support of clinical studies.

**Fully automated protein precipitation LC/MS/MS assay using a 96-well plate technology and track robotic system**

*Scott Paul Depee, GlaxoSmithKline, Research Triangle Park, NC, USA*
*Co-author: Brent T. Butler*

The need arose in our department to develop a faster and safer yet user-friendly means of sample preparation before sample analysis. With shorter mass spectrometer method run times, the rate-limiting factor was the sample-processing procedure. To expedite this procedure, automation through robotics and 96-well plate technology was implemented. Not only is this robotic protein precipitation assay process faster than manual preparation, but also the precision and accuracy are comparable. In addition, our staff has less exposure to potential pathogens in the samples, therefore increasing safety. This robotic system is operated by very user-friendly software that enables the processing of samples either in a fully or semi-automated manner. The system can manage 96 samples in $< 1.5$ h.

**Automated offline solid-phase extraction for polar new chemical entities using the Zymark Rapid Trace systems**

*Daksha Desai-Krieger, R. W. Johnson PRI, Springhouse, PA, USA*

A generic automated offline SPE method using the Zymark Rapid Trace( systems was developed for three very polar new chemical entities (NCEs) undergoing development at RWJPRI, namely a neuroaminidase inhibitor, a cephalosporin and a thrombin inhibitor. The polar characteristics of these compounds made it impossible to carry out liquid–liquid extraction as a sample clean-up step for these compounds. Our goal was to obtain simple automated SPE methods as sample clean-up for these polar compounds from biological matrices and develop quantitative LC-MS/MS assays for these compounds. Challenges encountered during assay development and details of the method will be presented. Transfer of these automated offline assays to online automated SPE and/or offline automated 96-well SPE will also be discussed.

**New technique for a high-throughput solubility assay**

*Dima Voloboy, pION, Inc., Woburn, MA, USA*
*Co-authors: Chau M. Du, M. Strafford, K. Tsinman and A. Avdeef*

We have successfully adapted the classical shake-flask method to the 96-well microtitre plate format to obtain near shake-flask-quality results at high-throughput speeds. The new instrument uses a robotic fluidics system and a parallel detection system employing a microtitre plate UV (190-500 nm) spectrophotometer. Samples are introduced as DMSO stock solutions.

The essence of the approach is based on making UV concentration reference solutions from molecules—especially ionizable molecules—whose UV spectroscopic properties are unknown at the start of the assay. This can be achieved in either of two ways, with or without cosolvents.

A unique computational method was developed to extract the aqueous intrinsic solubilities of drug molecules from data distorted by DMSO drug binding or drug–drug aggregation reactions.

An improved method for determining concentration by UV spectrophotometry was derived. It uses a novel peak-shape algorithm for adjusting weights in a whole spectrum-weighted regression analysis, matching spectra of reference solutions (of known concentration, under conditions avoiding or suppressing precipitation) to solutions containing an analyte of unknown concentration (due to precipitation).

A quality-assessment scheme has been developed around the standard drugs diclofenac, indomethacin, flurbiprofen, chlorpromazine, phenazopyridine, verapamil, piroxicam and griseofulvin.

The new solubility method, which allows determination at one pH or as many as 96 pHs, has a limit of detection of about $0.1\,\mathrm{mg\,ml^{-1}}$, as demonstrated by measurements of the intrinsic solubilities of terfenadine and tamoxifen.

About 200 assays may be performed per day on the instrument.

**Automated solution-phase synthesis workflow at Schering AG**

*Christoph M. Huwe, Schering AG, Berlin, Germany*

At Schering we have established a flexible, integrated solution-phase synthesis, purification and reformatting workflow based on Chemspeed- and Zymark-automated synthesizers, parallel normal-phase chromatography as well as reversed-phase HPLC-MS purification equipment, and a highly automated Zymark reformatting robot. In this presentation, the special features of the chosen equipment, the ideas behind the system design and the respective workflows will be discussed.

### Intelligent automation of HPLC method development

*Douglas R. Myers, Intelligent Laboratory Solutions, Naperville, IL, USA*
*Co-author: Jeffrey D. DeCicco*

Developing new HPLC methods is a time-consuming and knowledge-intensive task. We have developed an online software package called ChromSmart that automates HPLC method development for both normal and reversed-phase chromatography for chiral and non-chiral sample mixtures. Unlike other software packages that assist with offline HPLC modelling, ChromSmart uses online artificial intelligence technology to capture and implement the method-development knowledge of expert chromatographers. ChromSmart contains a real-time intelligent experiment planner and rule-based engine that execute the HPLC methods, monitor results and dynamically update the development plan. A complete audit trail of the decision-making and results is generated.

ChromSmart comes with a core set of chromatography knowledge that automates method development. Based on user preferences, such as retention times and resolution, ChromSmart develops a method-development strategy. This strategy is executed and can dynamically change based on information from online, real-time experimental results. Features include automated equilibration, detection and cleaning of retained components, gradient and isocratic optimization, peak purity analysis, and peak tracking. When ChromSmart finds a method that meets or exceeds the user's criteria, it notifies the user and continues to develop another method for the next sample. ChromSmart operates $24 \times 7 \times 365$ significantly increasing productivity of equipment and personnel.

### Laboratory automation using Zymark robots and UV fibreoptics from Delphian Technology

*Jeffrey B. Medwid, Wyeth-Ayerst Research, Pearl River, NY, USA*
*Co-authors: Jerry Edgar, Syed Rahman and Scott Keenan*

In the fast-paced pharmaceutical business, quicker turnaround times and larger sample loads face every analytical laboratory manager. Unfortunately, this must be accomplished with no significant increase in analyst resources. Laboratory automation is the only answer. Our research automation laboratory contains several Zymark robots including the Multidose Dissolution Workstations and Tablet Processing Workstations. The latest addition to our laboratory is a UV Fiber Optic Dissolution Workstation manufactured by Delphian Technology. This presentation will highlight some of our recent work accomplished using these workstations and some of our other initiatives in automation.

### 'Cradle to grave' tracking of synthetic combinatorial libraries

*Phil Small, Tripos Receptor Research, Bude, UK*

A process-integrating design, synthesis and analysis of combinatorial libraries has been implemented at Tripos Receptor Research. Crucial to the success of this process has been the development of a proprietary informatics system. This has so far been developed to manage reagent inventory, track samples and record data from synthesis and analysis to provide a valuable database of compound information.

The combination of Tripos' proprietary design software with automated synthesis and informatics leads to the production of drug-like libraries with well-defined purity and full synthetic history.

This presentation will cover what were considered the important aspects involved in setting up and managing an automated chemistry facility.

### Imaging technology in ultrahigh-throughput screening: overcoming the bottleneck of plate reader throughput

*E. Michael August, Boehringer Ingelheim Pharmaceuticals, Inc., Ridgefield, CT, USA*
*Co-authors: Lori Patnaude and Carol A. Homon*

Screening throughput is limited by its slowest step, and thus a major goal of uHTS must be to identify and overcome these bottlenecks. With the advent of high-density liquid-handling devices capable of delivering liquid to 384 or more wells at rates approaching 1 plate $min^{-1}$, the bottleneck has shifted from reagent delivery to plate reading. Conventional plate readers require several minutes to obtain data from an entire plate.

Imaging technology allows the detection of an entire plate simultaneously. Coupled with rapid, sensitive image-analysis software, such a system enables data capture to keep pace with reagent delivery. We present here screening results from a recent enzymatic Delfia uHTS campaign comparing the Wallac Viewlux Imaging system with the LJL Analyst. Several hundred plates in both 96- and 384-well formats were read on both readers and the data were analysed with a variety of statistical parameters.

Similar comparisons of other assay formats will be discussed. The inherent flexibility of imaging systems is highly compatible with assay miniaturization and the trend to higher-density plate formats. However, we must also realize that the bottleneck in the overall screening process has not been removed, but surely shifted to another step.

### Finally the paperless and compliant laboratory

*Ed Halpin, VelQuest Corporation, Hopkinton, MA, USA*

Compliance and cost have long been at odds in the modern regulated laboratory. Focusing on one meant sacrificing the other. Regulatory compliance today is a labour-intensive, paper-based operation. The 'paperless' laboratory has been a goal of the pharmaceutical industry for over the past decade. The confluence of technology and innovation has reached a point where the paperless laboratory is now economically and technologically practical. Good manufacturing practices and 21CFR Part 11 define the requirements to attain a paperless, compliant laboratory.

The solution would enable direct electronic data capture from human observations, instruments and networked PCs replacing paper notebooks, files and control sheets. The solution would enhance the collection of data and metadata by dynamically linking it to the procedure by which it was collected. Reviews, audits, investigations and approvals would be electronic processes, replacing the current paper systems. Redundant checking would be replaced by 'review by exception' and resources reallocated towards the real goal of bringing new human therapies to the market more rapidly. The solution must also complement and exchange data with other laboratory applications such as Laboratory Information Management Systems (LIMS) and Chromatography Data Systems (CDS).

### Generating predictive models from high-throughput profiling

*Dragos Horvath, 128 rue Danton, Rueil Malmaison, France*
*Co-author: Michael Entzeroth*

Current drug development is characterized by a strong belief in high-throughput methods, both in combinatorial chemistry and screening. The trigger for constantly increased performance is the need the industry to adjust to the race against time in order to secure shares on the market place. Over many years the industry has tried to keep pace by the constant introduction of new technologies. The associated costs for drug development increased over the last years. Aggregated R&D expenditures for new chemical entities (NCE) peaked in 1996 close to US$500m.[1] Recently, Lehman Brothers estimated the total costs for a launched product to account up to US$635m,[2] while for drugs entering clinical trials between 1972 and 1982 these costs were estimated to add up to US$312m.[3] Research-based companies will invest $26.4 billion in R&D in 2000, a 10.1 percent increase over 1999.[4] The number New Chemical Entities approved by the FDA has not changed significantly over the last years (1997: 39, 1998: 30, 1999: 35), indicating that the success rate is not related to the initial efforts.

The portion of IND applications that fail has been estimated to approximately 20% in phase II, 60% in phase II and most importantly 87% in phase I.[5] 83% of the drugs for which INDs were filed between 1964 and 1989 were dropped before reaching NDA status.[6,7] For the pharmaceutical companies each dropout during the development is associated with huge costs that reduce the profit derived from NCEs that reached the market. The failure of the candidates can be primarily attributed to efficacy, safety and economic concerns. The predominant reason for failure, however, are inappropriate pharmacokinetic properties of the drug candidate (39.4%), followed by adverse reactions and toxicity that add up to 21% of the causes for withdrawal from development.

In most of the cases the lack of a complete picture of the drug's properties is not available at the time the decision to select a respective candidate is taken. In part this is due to the current process of drug discovery where information is accumulated stepwise rather than in a defined parallel manner. The choice of the candidate taken forward is often dominated by emphasizing the aspect of potency rather than addressing early on selectivity and drug property issues. On the other hand, the companies have accumulated vast amounts of *in-vitro* and *in-vivo* data that may be used to refine the decision matrix either by retro- or prospective interpretation of existing results. As a consequence and in order to take the serendipity out of such fundamental decisions, many pharmaceutical companies have started broad range profiling (i.e. testing the drugs in parallel against a panel of different in-vitro model, both in pharmacology and ADME/PK) in the preclinical phase and have put emphasis on the development and automation of secondary tests. High throughput profiling using robotics and automated workstations allows to accumulate today a broader knowledge on the hits identified in the primary screening. Both pharmacological—specificity and selectivity versus other targets, such as enzymes and receptors—and pharmaceutical properties—physicochemical parameters or metabolic and in-vitro safety aspects—of the candidates are evaluated in depth.[8]

Through the progress in linking of high-throughput pharmacological, physicochemical and '*in-silico*' methods with in-vitro approaches, progress is being made in predicting drug properties.[8] The goal to predict *in-vitro* and, finally, *in-vivo* properties of drugs has become reasonable by linking the chemical structure via molecular descriptors, area descriptors or ComPharm fields to certain *in-vitro* profiles that again can be correlated to the corresponding in-vivo effects (figure 1).

An example for pharmacophore fingerprints are the Fuzzy Bipolar Pharmacophore Autocorrelograms (FBPA[9]) monitoring the numbers of atom pairs within each of the $252 = 21 \times 12$ categories that can be defined in terms of the 21 combinations of six pharmacophoric features $a,b \in \{$Hydrophobicity, Aromaticity, Hydrogen Bond Acceptor & Donor, Cation, Anion$\}$ times 12 considered distance ranges $\Delta \in \{(3..4), (4..5), \ldots, (14..15)\}$ [Å]. For similarity searches and structural comparisons, these fingerprints are extremely powerful in combination with ComPharm field descriptors[10] that are taken as the intensities of the empirically defined 'pharmacophore fields' generated by the atoms of the considered compound at the space points occupied by a reference structure, in a configuration corresponding to its optimal alignment with respect to this reference.

The data mining approach described in this report is part of the BioPrint™ project[11] that makes use of a broad spectrum profiling of marketed drugs and candidates in more than 90 different *in-vitro* models. The marketed drugs, included in the test collection, comprise more than 1,500 compounds that are available in the US pharmacies today. Important are also the drug candidates

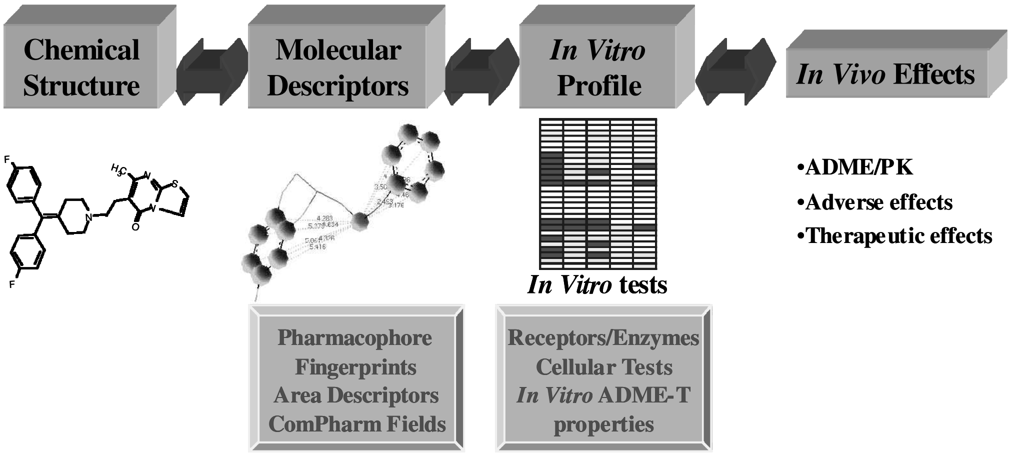

*Figure 1. Predictive modelling of the properties of compounds correlates chemical structures via molecular descriptors with ADME/K and therapeutic effects.*

included which have failed to make it all way through the different phases of preclinical and clinical drug development. The models and predictive tools generated open a new approach for candidate selection and early discovery activities, such as target identification or library design and will result in the long term in substantial cost savings due to reduced failure rates and time spent on the research and development process. *In-silico*, linear models to describe solubility and permeability as well as predictions in pharmacology based on predictive neighborhood behavior are presented.

### Linear ADME models

The interest in an in-silico logD prediction model resides both in its applicability in library design and its further use as a calculated molecular descriptor in other structure-activity relationships, if the activity under study is expected to relate to the overall lipophilicity of compounds. By contrast to logP models that mostly rely on incremental group contributions and/or molecular descriptors of the average polarity of the solvent-accessible surface (an aspect which is in our model successfully captured in terms of PTA descriptors), a logD model needs to account for proteolytic effects in the aqueous buffer of pH = 7.4 used for experimental determinations. In our models, proteolytic ionization of acids/bases is implicitly accounted for by the pharmacophore descriptors relying on rule-based assignments of a positive/negative charge status to ionizable groups. Furthermore, bipolar pharmacophore elements or field overlap terms successfully account for potential pKa shifts due to neighboring groups of the ionizable center.

### Linear apparent permeability models

The main challenge in predicting the apparent permeability of compounds through a Caco-2 cell monolayer is

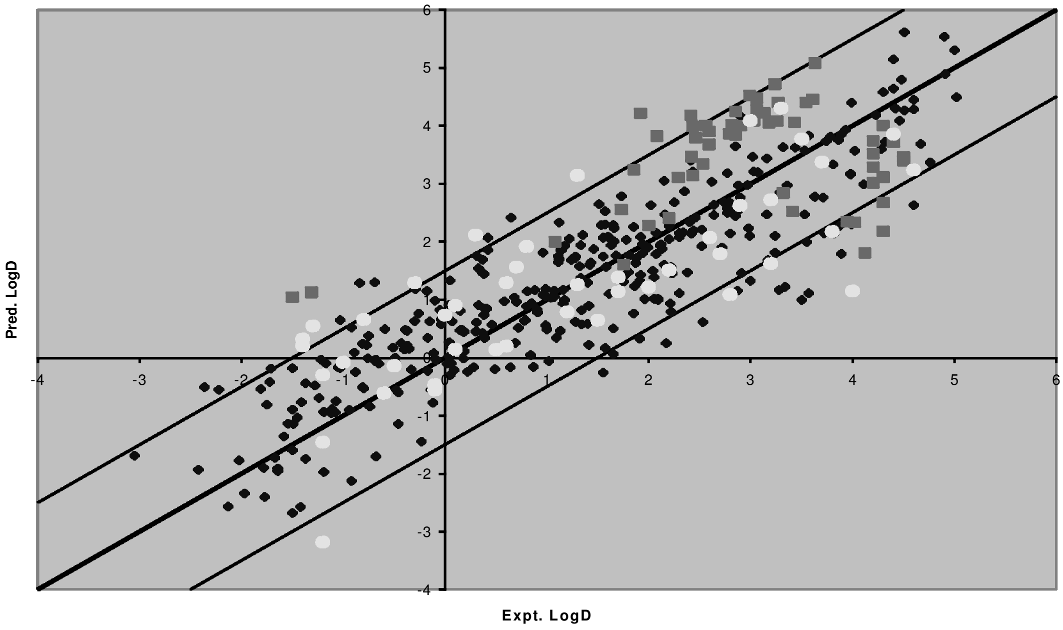

*Figure 2. Predicted versus experimental log* D. *The properties of compounds from a diverse library of combinatorial compounds (squares) and medicinal chemistry (circles) were calculated from a model based on a training set of 320 entities (rhomboids).*

the *in-silico* recognition of molecules which are transported by one of the active in- or efflux mechanisms. This will enable the introduction of specific correction factors explaining the difference between the actual membrane crossing rate and the rate expected on the basis of purely passive transmembrane diffusion. While the latter aspect of predicting the passive diffusion rates has been tackled in various publications, little progress has been made to understand the problem of transported/effluxed compounds, routinely discarded as 'expectable' outliers in previous work. By contrast, the herein described modeling effort focused on pharmacophore descriptors in order to search for potential pharmacophore motifs that may characterize efflux and/or transportation, while descriptors of the molecular surface properties and the generic calculated logD descriptors were supposed to account for passive membrane crossing properties. The calculated logD (or, even better, the experimental logD values) are relevant variables of the linear log($P_{A-B}$) apical-to-basal apparent permeability model. A positive coefficient for the linear contribution, together with a negative term in $logD^2$ characterize an optimal logD range maximizing the passive membrane crossing rate. This makes physical sense, since too hydrophilic compounds never leave the aqueous phase, while too hydrophobic ones tend to accumulate in the membranes.

In spite of the important number of bipolar pharmacophore elements entering the model, a series of heavily effluxed compounds are nevertheless mispredicted by the model and appear as 'false permeable'. Most of the outliers in the marked area are shown (figure 3, circled area) to be indeed effluxed molecules, since their measured basal-to-apical apparent permeabilities were significantly higher that the corresponding apical-to-basal values.

It can be therefore concluded that either (a) efflux cannot be understood in terms of bipolar pharmacophore descriptors only or (b) efflux might in principle be characterized in terms of such descriptors, but the number of descriptors that would be required to enter the model is much larger than the number of examples of effluxed compounds currently included in the data set—note that multiple efflux/transportation mechanisms with potentially different pharmacophore characteristics coexist in Caco-2 cells, and that enough compounds exemplifying each one of them would be required to solve the problem.

The introduction of ComPharm descriptors overriding the limitations of bipolar pharmacophore terms is indeed seen (figure 3) to significantly improve the permeability prediction of the outliers from the previous model. These field descriptors were obtained on the basis of superimposition models of BioPrint$^{TM}$ compounds against some of the most heavily effluxed/transported substances encountered in this set.

### Solubility model

A categorical solubility model based on a linear equation has been developed (figure 4) in order to predict the solubility class of the compounds: (1) Low: $S < 10\,\mu M$; (2) Medium: $10\,\mu M < S < 100\,\mu M$; (3) High: $S > 100\,\mu M$. This model (not shown here) included 12 explaining variables, such as logD and $logD^2$ and various Pharmacophore Type Areas (PTA) and bipolar pharmacophore elements that predicts drug solubility from its structural features. With encouraging results it has also been compared with other solubility prediction software packages and shown its advantages with 86% of the drugs predicted in the correct solubility class.

### Predictive neighborhood behaviour (PNB) models

By contrast with linear approaches that require a specific equation for each of the properties that are to be predicted in terms of molecular descriptors, PNB models rely on the measured properties of related compounds (e.g. nearest neighbors in descriptors space) from the

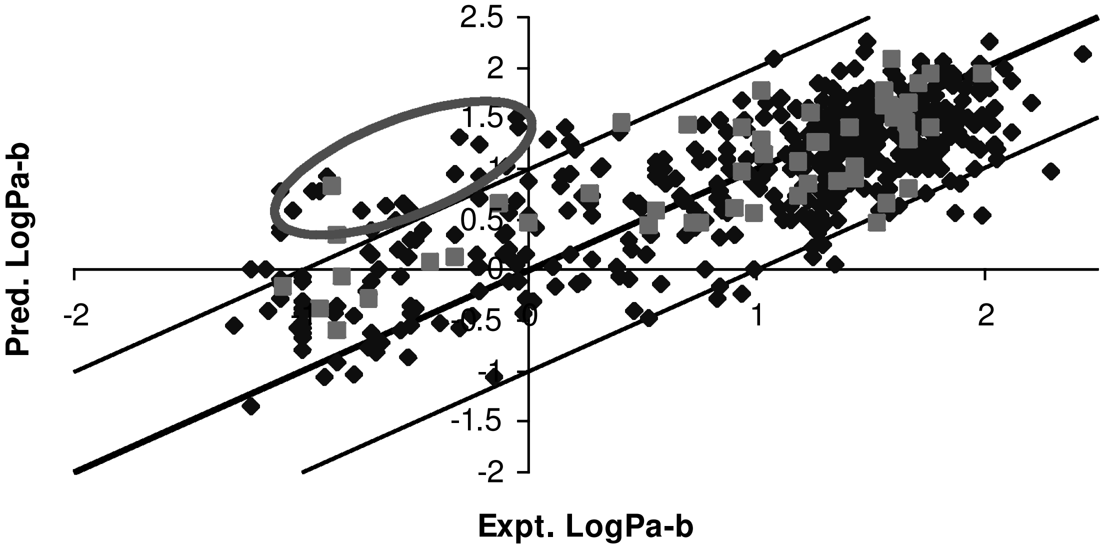

*Figure 3. Improvement of the permeability prediction of effluxed compounds due to the use of ComPharm field descriptors in the model. Training set (rhombs); test set (squares).*

| Expt. Class -> | H | M | L | Tot.Pred |
|---|---|---|---|---|
| **Pred. Class:H** | **265** | **19** | **2** | 286 |
| **Pred. Class:M** | **15** | **15** | **12** | 42 |
| **Pred. Class:L** | **0** | **0** | **12** | 12 |
| Tot.ExptClass | 280 | 34 | 26 | 340 |

*Predicted in correct class (green)=(265+15+12)/340=86%*

*Predicted in neighboring class (yellow)=(15+19+12+0)/340=13.5%*

*Predicted in WRONG class(red)=2/340=0.5%*

*Figure 4. Comparison of the predicted solubility class (vertical) of compounds versus the experimental class (horizontal) using the BioPrint™ solubility model.*

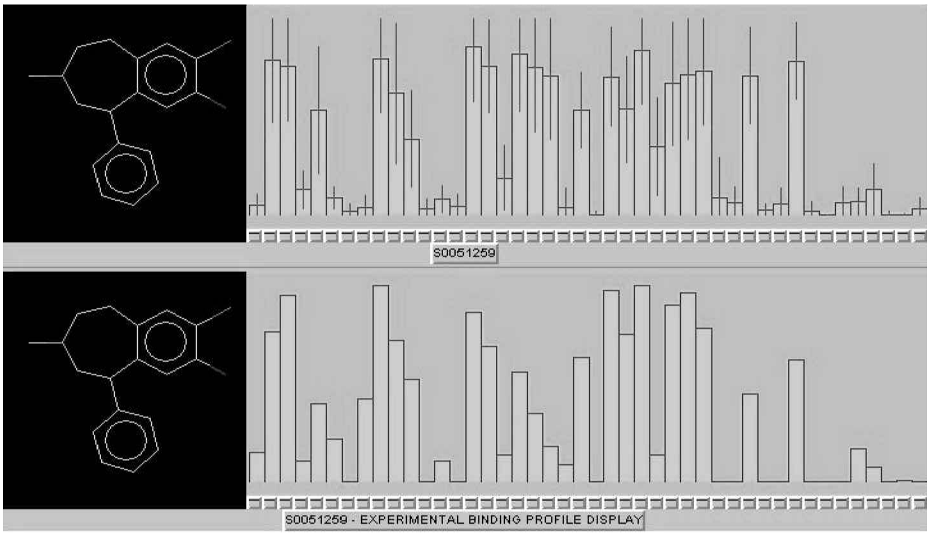

*Figure 5. Receptor profile of SCH23390, 10 µm. (upper) Predicted profile (each bar represents the percentage inhibition in a pharmacological model + RMS prediction error) based on the activity of its nearest neighbours; (lower) experimental data in the same assay panel.*

BioPrint set in order to provide an estimation of the property value of a new molecule. The PNB algorithm works in principle like a 'fuzzy data base querying tool' in the sense that if molecule X is not included in the BioPrint database, the BioPrint compounds that are most similar to X are retrieved and used for the evaluation of the properties of X. The property of the unknown molecule is computed as a weighted average of the experimentally determined properties PN(x) of its nearest neighbors N(X) from the BioPrint set, where the weighing factors are decreasing functions of the molecular dissimilarity scores that define how 'near' a neighbor N(X) is with respect to X. These dissimilarity scores are Dice scores calculated on the basis of FBPA, PTA and EFO descriptors, the intervening empirical parameters being specifically fine-tuned in order to maximize the predictive power with respect to a particular property. Figure 5 shows the example in the case of SCH23390, a D1 receptor ligand, which was not included in the original data set, however, tested later for comparison.

### Conclusion

During the last decade technologies such as ultrahigh-throughput screening and combinatorial chemistry have significantly contributed to the advances in drug discovery. The increase in R&D costs associated and the bottlenecks further down the development as well as the high dropout rates have increased the demand for an alternative approach. BioPrint™ combines molecular descriptors, high-throughput profiling with *in-vivo* drug profiles. It takes advantage of data mining technologies to generate predictive models that link the three components. These models help to build a effective strategy that will foster drug discovery in the new millenium.

### References

1. Kettler, H., 1999, *J. Comm. Biotech.*, **6**, 60.
2. Kettler, H., 1999, *Updating the Costs of a New Chemical Entity* (London: OHE).
3. DiMasi, J. D., Hansen, R., Grabowski H. G. and Lasagna, L., 1991, *J. Health Econ.*, **10**, 107.
4. PhRMA., *Pharmaceutical Industry Profile 2000*, 20.
5. Kennedy, T, 1997, *Drug Disc. Today*, **2**, 436–444.
6. DiMasi, J. A., 1995, *Clin. Pharmacol. Ther.*, **58**, 1.
7. Prentis, R. A. and Walker, S. R., 1986, *Br. J. Clin. Pharmacol.*, **21**, 437.
8. Smith, D. A. and van de Waterbeemd, H., 1999, *Curr. Opin. Chem. Biol.*, **3**, 373.
9. Horvath, D., 2001, in A. K. Ghose and V. N. Viswanadhan (eds), *Combinatorial Library Design and Evaluation—Principles, Software Tools and Applications in Drug Discovery* (New York: Marcel Dekker), 429.
10. Horvath, D., 2001, in M. Diudea (ed.), *QSPR/QSAR Studies by Molecular Descriptors* (New York: Nova), 395.
11. Entzeroth, M., Chapelain, B., Guilbert, J. and Hamon, V., 2000, *JALA*, **5**, 69.

### Powder-dispensing capabilities to the Zymark Benchmate system

*Erin C. Heritage, R. W. Johnson Pharmaceutical Research Institute, Raritan, NJ, USA*

One of the most fundamental routines for a chemist is the preparation of samples from dry powdered samples. There have been many expensive attempts to automate this process. We demonstrate powder dispensing using an existing robot workstation and some common disposables.

Powder dispensing was accomplished by building a dispensing station on an integrated weighing robot—the Zymark Benchmate. The operating principle of the station is based on applying a vacuum to an airline connected to a pipette tip. The system is designed to transfer dry powder from four-dram vials to 16 × 100 test tubes. The user can specify target weight and replications for each compound through a custom-designed Visual Basic program.

The sample vial is basically carried to the fixed location of the pipette tip, placing the tip into the powder. The vacuum is applied to the airline and the vial removed. The vacuum holds a small amount of sample powder on the pipette tip. A test tube is then moved into position below the pipette tip, the vacuum is released and the powder is dispensed into the 16 × 100 test tube. When the target weight is reached, sample preparation (e.g. dilution to concentration and mixing) can then be accomplished on the same workstation.

### uHTS of phosphatases using 2D-FIDA anisotropy detection

*Flora Tang, Sugen, Inc., South San Francisco, CA, USA*

Various parameters of fluorescence measurement, such as translational diffusion, brightness and rotation, have been widely recognized as readout fully amenable to homogenous, miniaturized uHTS. A novel detection system using Evotec's FCSplus reader, which combines the fluorescence-intensity distribution analysis (FIDA) anisotropy detection with confocal microscopy, provides a very sensitive and ultrahigh-throughput assay format. The collaboration between Sugen and Evotec OAI is focused on phosphatases and has demonstrated successful adaptation of 2D-FIDA anisotropy detection for phosphatase assays at the 1 μl/well level. A discussion of this assay format and the uHTS screening results on the automated EVOscreen Mark II system for several phosphatases will be covered.

### Considerations in dissolution automation

*G. Bryan Crist, VanKen Technology Group, Cary, NC, USA*

Among the future challenges for the pharmaceutical laboratory exists the necessity to automate dissolution methods to allow maximum throughput with optimum compliance. This presentation will provide suggestions for determining the level of automation needed to obtain peak performance as well as providing insight into regulatory compliance issues. Associated topics to be discussed include: novel dosage forms today and providing analytical challenges in the future, alternate methods of analysis, the dissolution apparatus of the future and the involvement of new analytical approaches. Instrumentation flexibility will be required in the dissolution laboratory of the future to utilize fully pooled sampling techniques, online UV, online HPLC, capillary electrophoresis and even atomic absorption for nutritional analysis.

Semi-automation to total automation concepts including *in situ* methods of analysis will also be discussed. As novel concepts evolve each day, engineers working with scientists will lead to more efficient, better-integrated dissolution instrumentation in the future.

### Update from PQRI's Blend Uniformity Working Group: balancing workload with batch homogeneity assurance

*Garth Boehm, Purepac Pharmaceutical Co., Elizabeth, NJ, USA*

The Product Quality Research Institute (PQRI) is a consortium between industry, academia, and FDA that aims to provide a scientific basis for the development of regulatory policy. One of PQRI's first initiatives was to form a Blend Uniformity Working Group (BUWG) charged with providing a scientific basis for continued development of FDA policy on BU testing. The BUWG sought wider industry input through a survey of current BU testing practices and a public workshop. Using this input together with the experience of the BUWG members and colleagues, a draft proposal was developed and refined. It provides a guide for appropriate testing during scale-up and validation, and for subsequent routine manufacture. The proposal is based around initially establishing the relationship between BU testing and stratified testing of the resulting dosage units. The type and amount of testing recommended during routine manufacture depends on the outcome and robustness determined from the validation testing. The BUWG is currently seeking data from industry to challenge the proposal with real data to establish the validity of the suggested approach. The next step will be to seek public comment on the BUWG proposal.

### Potential impacts of implementing automated dissolution

*Gerard Schneider, LEC Consulting, Blairstown, NJ, USA*

The Sotax AT-70 Smart offers the ability to automate fully both USP Apparatus 1 and 2 dissolution methods. This system, used in conjunction with other efficiency-driven technologies such as premanufactured medium and online spectrophotometric analysis of samples, can profoundly reduce the resources required for conventional dissolution testing while enhancing the consistency of results. Most importantly, these efficiency gains can be achieved within the parameters of a compliance-conscious industry.

The effects of implementing such technology will be discussed. The discussion will address the effect that efficient, automated dissolution testing has on the product development area, where companies are focussed on delivering products to market in increasingly less time, as well as marketed product stability and release laboratories where QC managers are asked to reduce sample turnaround times without increasing resources. Examples of efficiency gains will be given in both cases.

### Assay automation: let humans do what they are good at and take robotics to the limit

*Gladys Range, Human Genome Sciences, Rockville, MD, USA*

In the automation of any process, there are basic steps that when carefully suited and applied to a particular laboratory assay or workflow, serve as a tool for effective automation. Biological validation, time and high-throughput screening assays are, by nature, very different, and are designed with different criteria in mind. A biological assay is a complex system designed to yield biological activity using qualitative measurements. Issues of cost, amount and quality of reagents, although important, do not play a paramount role in development. The biological assay is usually a manual assay that requires significant change before it is automation ready.

High-throughput assays in contrast are specific, fast, reproducible, low standard deviations, low cost and usually can be automated as much as mechanically and humanly possible.

Key elements in assay automation are sample sourcing, screening design, robotics' hardware and data management. These elements need individual careful analysis, a master plan and follow-up. A challenge in automation is to enable individual laboratory personnel to contribute to the fine ongoing activity of building a database by doing their work without stopping to write in a laboratory book or even to enter information in a computer. These in return will prevent redundancy and will improve accuracy and completeness.

Second-generation laboratory robotics and liquid handlers are now designed to handle very high-throughputs, very dense formats and very small-volume applications. Likewise, the recent development of sophisticated fluorescence systems operating at longer wavelengths, using time-resolve signals, and fibreoptics' technology reduce read time and facilitate moving away from less desired chemistries and separation steps. While some of the very large complex robotics systems could automate the complete sequence of operations comprising an assay, a higher level of productivity is achieved by paying careful attention to the workflow. Determining the optimum assay conditions, standardizing some of the operations and selectively introducing automation bring in the best results and benefits.

### Improving the quality and speed of automated sample transfers

*Glenn Smith, GlaxoSmithKline, Research Triangle Park, NC, USA*
*Co-authors: Jimmy Bruner, Charles Buckner and Bob Biddlecombe*

The quantitative transfer of biological fluids is the most critical aspect of automated sample preparation. The sampling of plasma, serum, blood or urine represents the most difficult and time-consuming step in most bioanalytical extraction procedures. We will present two practical solutions that address these separate problems:

- automating 'difficult' sample transfers with higher quality;
- significantly increasing the processing speed of most 'simple' sample transfers.

First, most laboratories automate sampling on a multiple-tip liquid-handling workstation such as a Packard MultiPROBE® or Tecan Genesis™. Today, samples are most commonly transferred from various size tubes to a deep-well plate or a 96-well solid-phase extraction (SPE) block. Despite their widespread use, these systems cannot perform sampling with sufficient precision and accuracy for certain applications (e.g. samples containing clots or other insoluble matter; samples with low or insufficient volumes).

We will show how the integration of an analytical balance on an *x-y-z* liquid-handling deck can easily improve the overall accuracy of assay data. The application also allows the workstation to process samples that would otherwise need to be transferred manually. Samples are aspirated in parallel then dispensed one at a time, allowing the balance to capture the exact weight (=> volume) of each sample that was added to a deep-well or SPE plate. The data are used to correct automatically for any differences in sample weights, usually by pasting the results directly in the 'dilution factor' column of an analytical system's worklist.

Second, for most other applications in which reliable transfers are easily achieved without gravimetric confirmation, the speed of sample processing remains a rate-limiting step. Most systems require about 20–45 min to transfer 96 samples. This time is significant and limiting compared with the 1–2 min required for other steps such as the addition of a reagent using a 96-channel pipetting station (e.g. Zymark RapidPlate™-96, Tomtec Quadra 96™, Apricot PP550™).

We will detail how custom sample racks can be used to speed up tube-to-plate sample transfers dramatically.

These novel racks have a standard microplate footprint and can be used on any 96-channel pipetting station. The racks, which consist of complimentary pairs, each hold 48 sample tubes arranged in an alternating (staggered) sequence. With these racks, 48 samples are transferred simultaneously and 96 are transferred in as little as 2 min. The racks work with most brands of 13-mm outer diameter cryotubes and 11-mm outer diameter microcentrifuge tubes commonly used in bioanalytical laboratories.

With these two inexpensive workstation modifications we can now automate 100% of our applications and significantly speed up the majority of these assays.

### Identification of cytokine-regulated genes associated with infertility and obesity by DNA gene-chip micro-arrays

*Grace Wong, Serono Reproductive Biology Institute, Randolph, MA, USA*
*Co-authors: H. Yarovoi, Q. Chen, S. Nataraja, Q. Xu, C. Liu, W. Mesadie J. Lai, J. Straaubhaar, J. Strickler, M. Tepper, M. Dreano, G. Garotta, S. Fumero, T. Wells, A. Eshkol and S. Arkinstall*

Although the sequence of the human genome has been determined, the regulation and function of many genes remain largely unknown. Cytokines are tightly regulated soluble proteins transiently produced by cells in response to immunological stimulation or disease manifestation. The expression of these proteins has been linked to many diseases including cancer, AIDS, obesity, autoimmunity, immunodeficiency and infertility, suggesting that cytokines, their receptors, as well as cytokine-induced genes are potential targets for new drug development. Microarrays such as gene chips and protein arrays are high-throughput technologies for drug discovery in the post-genomic era.

We employed DNA transcriptional profiling micro-arrays to detect genes under control of cytokines in selected experimental models. Hence, in ovarian granulosa cells, TNF stimulates DNA synthesis but inhibits FSH-induced oestrogen production. These results suggest that identification of TNF inducible 'master control genes' may reveal new targets for infertility and possibly other diseases associated with oestrogen. TNF induces more than 70 genes in rat ovaries *in vivo*, including genes for cytokines, kinases, receptors, transcription factors and enzymes. In contrast to its inhibitory effect on oestrogen production in the ovary, TNF stimulates production of this sex steroid in human pre-adipocytes and adipocytes derived from more than 10 patients. Interestingly, human pre-adipocytes/adipocytes themselves produce TNF as well as other cytokines and our data suggest a positive autocrine/paracrine mechanism for control of oestrogen production by fat cells. In addition to TNF, 16 cytokines including LT-a, IL-1, IL-6, IL-11, LIF and oncostatin M all stimulate oestrogen production in human fat cells.

Other cytokines such as TGF-$\beta$1, TGF-$\beta$2, VEGF, Rantes, IL-4, IL-10, IL-12, IL-18, CD40 ligand and TNF-related proteins such as LT-b, TWEAK, RANK Ligand, APRIL and BAFF have no inducing effect. These results indicate that TNF has differential and cell-specific actions on key endocrine mechanisms. Identification of genes induced or inhibited by TNF and other cytokines may lead to the discovery of novel targets for drug development.

### CrystalScreen: a novel microplate for automated protein crystallization

*Gunther Knebel, Greiner Bio-One GmbH, Frickenhausen, Germany*
*Co-authors: Lajos Nyársik1, Patrick Umbach, Martin Horn, Thomas Przewieslik, Wolfram Saenger, Hans Lehrach, Peter Opfermann and Holger Eickhoff*

The three-dimensional structure of any protein plays a key role in the process of understanding the exact function of these macromolecules. One proven approach to structural information of proteins is based on X-ray diffraction of single crystals. In the past, automated crystallization was restricted because a reliable hardware platform and well-suited microplates for high-throughput screening were missing.

The Max-Planck-Institute for Molecular Genetics (MPIMG), the Protein Structure Factory (PSF) and Greiner Bio-One have collaborated to develop a unique 96-well protein crystallization microplate (CrystalScreen™) with a standardized microplate footprint for high-throughput applications. Each of the 96 mother liquor wells corresponds to three crystallization wells. This allows checkerboard screening with up to 288 crystallization options per plate to investigate optimal crystal growth followed by 3D-structure analysis. In combination with a preformed lid, this microplate enables high-throughput multichannel screening of proteins in automated systems.

The system in use at MPIMG and PSF allows simultaneous sitting-drop and hanging-drop vapour diffusion crystallization experiments at reduced costs. A huge storage system for 10 000 crystallization microplates and a pipetting device based on solenoid inkjet technology enables one to set up a complete plate with 96 crystallization conditions in $< 3$ min. All crystallization wells are inspected in regular intervals with an automated camera-based detection system to inspect the crystal growth.

### Seamless integration of information in a pharmaceutical development environment: integrating technology from LabWare, NuGenesis, VelQuest and Waters

*Guy Talbot, Purdue Research Center, Ardsley, NY, USA*

This presentation will describe Purdue Pharma's approach to an integrated solution between Laboratory Information Management Systems (LIMS), Chromatography Data Systems (CDS), Process Management and Compliance Systems (PMC) and Scientific Data Management Systems (SDMS). It will describe the vendor-selection process and how Purdue Pharma created an environment to produce a best-of-breed information solution to creating the paperless laboratory. Working towards the creation of a paperless laboratory has the potential to result in significant benefits including resource liberation and an electronic compliance platform for process management and data review.

This paper will describe the process of working with four vendors to create a complementary data-management solution to Purdue Pharma's needs. The process of logging in samples, creating worklists, executing analytical methods, capturing data electronically, storing electronic records, and submitting reports will be reviewed. After data creation, electronic data review will be discussed enabling electronic 'instant replay' of laboratory information.

The primary benefits of this complementary solution approach hold the following possibilities.

- Increased capacity by liberating valuable resources from rework loops and the manual data review process.
- Leveraging the approach to validation of the overall solution saving time associated with multiple computer validation plans and execution.
- Promise of reduced time to market for new drugs by using a paperless environment where possible.

This presentation will describe the challenges, successes and experiences surrounding the task of integrating complementary information systems in a pharmaceutical development environment.

### Using laboratory automation to prepare compounds precisely for HTS

*Haissam Abdelhamid, Purdue Pharma LP, Cranbury, NJ, USA*

Pharmaceutical companies are constantly expanding their compound inventory and reorganizing their screening plates in an effort to ensure new chemical entities can be identified as rapidly as possible. Whether these new compounds come from libraries (focussed or diverse) or medicinal chemistry efforts, robotics has become an essential tool when preparing compounds for high-throughput screening (HTS). Using robotics to automate routine laboratory tasks not only increases the rate at which these activities can be completed, but also reduces incidents of human error. In our laboratories, we have integrated several robotic systems to aid in compound dissolution and HTS plate preparation. These enhancements have saved time and increased the reliability of our screening inventory, particularly when coupled with our in-house data-management and inventory tracking software. Examples of laboratory automation used at Purdue over that past year will be presented.

### DNA micro-array fabrication and processing: automation in the laboratory

*Katrine Verdun, BIOGEM, Division of Biology, University of California San Diego, La Jolla, CA, USA*
*Co-authors: Richard Rouse and Gary Hardiman*

The confluence of robotics, biotechnology, computer sciences and the completion of genome sequencing efforts for several organisms have resulted in revolutionary changes in how biomedical research is carried out. It is now possible to fabricate high-density arrays of specified DNA sequences that include every known gene of an organism on a single glass slide. Labelled RNA or DNA targets (such as mRNAs obtained from cells, tissues or organisms under different conditions) can be analysed by hybridization on the array. Differences in the levels of expression for thousands of genes can thus be assessed all at the same time in a single, simple experiment. Genomics, informatics and automation are playing increasingly important roles as discovery tools in the basic biological sciences, and as diagnostic and rational therapeutic aids in the clinical arena. We discuss the use of automation to increase productivity in micro-array fabrication and describe how automated procedures increase the quality of results in micro-array experimentation.

### Use of the Bio-Tek Precision 2000™ automated pipetting system for micro-array sample preparation

*Paul Held, Bio-Tek Instruments, Winooski, VT, USA*
*Co-author: Gavin Picket*

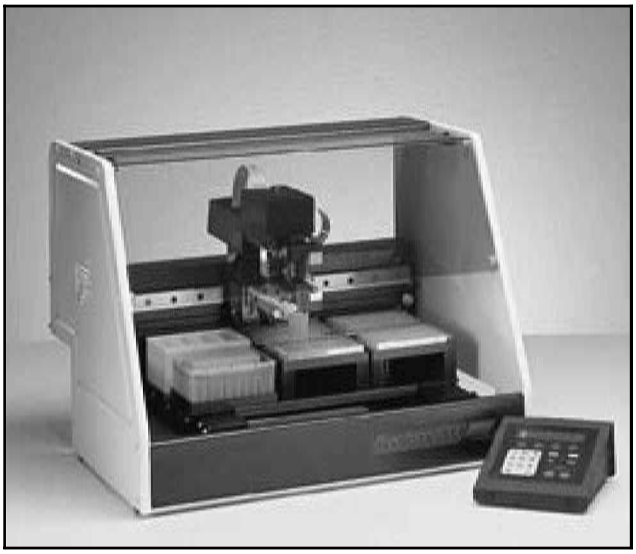

The production of micro-arrays requires the spotting of large numbers of unique DNA fragments onto several different substrates. While several commercially available instruments have automated this spotting task, the sample preparation, culture propagation and maintenance of the DNA library are often performed manually with multichannel pipettes in 8 × 12, 96-well, or 16 × 24, 384-well formats. Manual multichannel pipettes, while more efficient than single-channel pipettes, still represent a large amount of pipetting with many opportunities for pipetting errors. Here we describe the use of the Precision 2000™ automated pipetting system to carry out many of the necessary pipetting tasks required for the preparation of samples for micro-array spotting. These steps include: the propagation of plasmid libraries, PCR reaction preparation, treatment of PCR products for agarose gel electrophoresis and the reconstitution of lyophilized samples before micro-array spotting. The Precision 2000 has a completely configurable six-station platform to hold the required pipette tips, reagent troughs and microplates (96 and 384 well) for fluid transfer. The platform is removable, allowing for multi-user friendliness, easy cleaning and set-up of the instrument. The eight-channel pipette arm moves up and down as well as side to side, while the platform moves front to back to provide complete access to all locations on the work platform and complete configurability. The pipette arm uses a proprietary technology to pick up and seal reliably any standard tip with individual free-floating barrels that compensate for tips out of position. An optional rapid dispense eight-channel manifold, which uses a precise bidirectional syringe pump to dispense accurately and rapidly fluids from a large unpressurized reservoir, is also available. The Precision 2000 has a built-in microprocessor that controls all movements. The flexible software, both onboard and PC-based, provides complete programming capabilities. For more complete automation, robotics interfaces can be developed using ActiveX® software commands. The Precision 2000's small size, with a 15 × 21-inch footprint and a height of 16 inches, allows it to be used almost anywhere including most biological safety cabinets or chemical fume hoods.

### Transposition and validation of a manual method of sample preparation on the TPWII

*Hélène Brillard, Novartis Pharma, Orléans la Source, France*
*Co-authors: Laurent Frances, Florence Dupas, Samir Haddouchi and Olivier Garinot*

The Analytical Development Laboratory NOVARTIS that works conjointly with the Pharmaceutical Development Department had recently acquired the Zymark Tablet Processing Workstation, TPWII.

Within this laboratory, analytical methods are developed to perform manual analysis. Consequently, we developed a procedure to transfer successfully a manual analytical method to this robotic system. First, the whole parameters of the TPWII that have an influence on sample preparation have been determined and, second, the most important ones have been pointed out in order that they be validated. This has led to the establishment of a transfer protocol, which is in two parts:

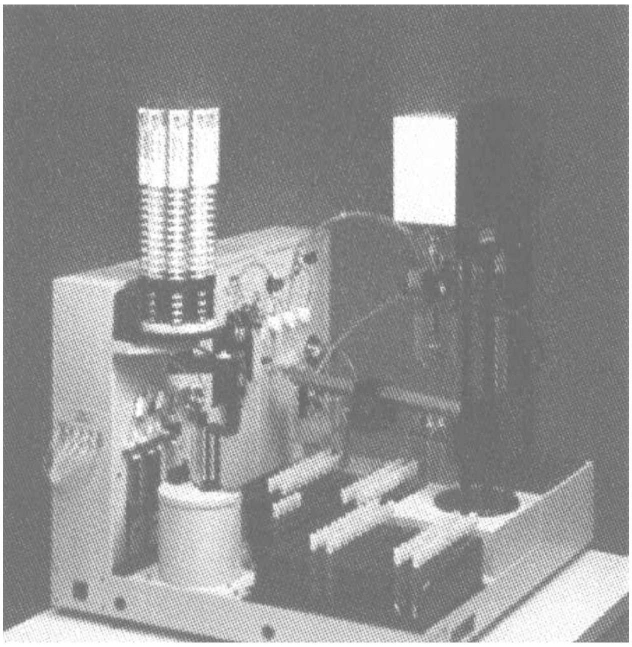

- development of the automatic method (study and optimization of all parameters);
- validation of the automatic method.

### Accelerating knowledge transfer for improved lead-candidate selection

*John P. Helfrich, NuGenesis Technologies Corporation, Westborough, MA, USA*

Today's modern drug-discovery and development research groups are expected to improve the efficiency of delivering NCEs to the clinic. The new high-throughput processes are dramatically increasing the raw number of data sets that must be interpreted for decision support across a global research team effort. The NuGenesis SDMS database serves as the centralized repository for analytical reports, compound presentation or summary documents, project reports, and instrumental raw data. This database can then be extracted to work in conjunction with your LIMS, Enterprise Data Management System and/or local specialized visualization and statistical data software products. If your data sources save or print, the NuGenesis SDMS platform can get it, automatically save it and allow fast efficient utilization across the entire enterprise. After all, the data become information that when effectively communicated turns into critical-path knowledge for decision support in drug discovery.

### Maximum return (strategic investments for high-throughput screening)

*James LaRocque, Wyeth-Ayerst Research, Pearl River, NY, USA*

Advanced technologies theoretically enable modern HTS laboratories to screen expanding libraries quickly against a drastically longer list of targets while reducing reagent costs and maintaining more rigorous quality of results. Even in large pharmaceutical corporations, however, the collision of fiscal reality and the cost of state-of-the-art HTS technologies make getting the most out of capital investments a critical skill for success. Wyeth-Ayerst Research has made cost-effective investments in Packard CCS PlateTraks and Wallac CCD imagers, while also continually upgrading our Zymark-integrated systems with new components and detectors.

This combination of equipment provides maximum throughput for 384-well formats and supports the transition to 1536-well formats with relatively modular units that can be programmed for multiple tasks. While the management of individual screening projects is delegated to individual scientists, the complexity of the automation infrastructure offers ample opportunity for the development of specialty skills, creating a well-rounded HTS staff. By practising both individual initiative and cooperativity, a relatively small group is empowered to achieve effective sample plate replication, 384- and 1536-well HTS, and rate-based hit characterization.

### Automated LC/MS analysis of biomolecules using ProMass

*Jeff Whitney, Novatia, Princeton, NJ, USA*
*Co-authors: Mark E. Hail and David J. Detlefsen*

The recent increase in genomic and proteomic discovery has increased the need for high-throughput automated tools for biomolecule characterization. The flood of new protein targets will ultimately demand more efficient tools for the evaluation of expressed proteins for drug discovery and structural biology studies. ProMass is an automated biomolecule deconvolution and reporting program used to process LC/ESI/MS data or single ESI mass spectra to derive molecular mass information. We recently integrated ProMass with the ThermoFinnigan Xcalibur data system to create a version of ProMass known as ProMassXcali™. ProMassXcali processes entire Xcalibur sample sequences, deconvolutes mass spectra from LC/MS data and produces web-based reports.

ProMassXcali uses a novel cross-platform deconvolution algorithm known as ZNova™. ZNova incorporates a unique charge-state scoring method that assigns the charge states of all signals in the ESI mass spectra and transforms the input ESI mass spectra to produce zero-charge mass spectra (i.e. molecular mass information). ZNova incorporates signal-processing techniques and unique logic that allow application to low charge state spectra and data of low signal-to-noise ratio. As a result, ZNova can be used to process data from a wide variety of biomolecules including large proteins, oligonucleotides, peptides, etc. In this poster, an overview of the ProMassXcali/ZNova system will be presented along with selected applications which highlight its utility in a high-throughput environment.

### Automated metabolite confirmation and identification using LC/MS and intelligent chemometrics

*Jeff Whitney, Novatia, Princeton, NJ, USA*

In recent years, drug-discovery researchers have placed greater emphasis on obtaining qualitative measures of drug candidate 'quality' by measuring ADME/Tox tendencies earlier in the discovery process. This has led to the development of *in-vitro* assay methodologies for measuring metabolic stability, cell permeability, solubility, toxicity, etc. The challenge today is to automate fully all aspects of these assays by integrating intelligent data analysis and interpretation tools into one simple-to-use solution.

This talk will focus on the analytical methods and intelligent data analysis approaches we have employed to assess rapidly metabolic stability and metabolite identification. With the use of SmartLCMS™ technology, we will demonstrate the rapid assessment of metabolic stability of a compound followed by automatic detailed re-analysis using on-the-fly intelligent data-analysis techniques. In addition to up-front intelligent automation, we will illustrate the preliminary results of our MetLab™ software suite for back-end chemometric-based data processing techniques to confirm automatically expected and unknown metabolites.

### Development of a high-throughput biochemical profiling platform

*Jeffrey Murray, Paradigm Genetics, Inc., Research Triangle Park, NC, USA*
*Co-authors: Ioana Popa-Burke and Chris Beecher*

Paradigm Genetics, Inc., has industrialized the process of gene-function discovery for human health, nutrition, crop production and industrial products. The company has designed the GeneFunction Factory™, an industrial-scale laboratory that explores the function of genes in organisms by integrating state-of-the-art sequencing technology with phenotype, metabolite and gene-expression profiling to collect hundreds of data points through a single technology platform.

The Metabolic Profiling group is responsible for monitoring changes in the biochemical profiles of organisms that occur over the course of development in response to stress or induced genetic modifications. This is accomplished by using LC-TOF-MS, GC-TOF-MS and ICP-MS instruments. We describe here the innovative high-throughput processes developed for the cataloguing, storing in a dry environment, grinding, dispensing and extraction/derivatization of samples using custom robots, as well as proprietary instruments such as the 'Mash-A-Matic and 'Buster'.

### Role of automation and robotics in high-throughput ADME profiling of potential drug candidates

*Kelly M. Jenkins, Bristol-Myers Squibb Co., San Diego, CA, USA*
*Co-authors: Robyn A. Rourick, Reginald Angeles, Marianne Teopaco-Quintos and Daniel B. Kassel*

Early determinations of pharmaceutical properties can serve as predictors of a compound's likely developmental success. Our laboratory has implemented high-throughput ADME assays that address absorption, metabolism and physicochemical properties in an effort to minimize discovery to market attrition. While trying to meet the throughput demands of parallel synthesis, we established an integrated solution for ADME assays which incorporates a SAGIAN™ core system for the determination of both metabolic stability in human liver microsomes (HLM) and cytochrome P450 (CYP) inhibition. This automated solution has allowed an increase in capacity, throughput and reliability for both ADME assays.

The HLM assay uses a Multimek™ 96-channel pipettor for liquid handling. The analysis plates are transferred offline for final analysis using high-throughput parallel LC/MS. The CYP inhibition method uses a combination of liquid handlers and a fluorescence plate reader to perform a single concentration profile assay for 88 compounds. CYP inhibition is measured for both CYP3A4 and 2D6 isozymes.

This system represents a fully integrated approach in support of high-throughput ADME evaluation in a drugdiscovery environment. The core system concept creates a plug-and-play approach that combines a series of modular stations to build a robotic system, which is flexible, upgradable and easily reconfigurable when assays change or are newly developed. The application of these strategies as a means of assessing metabolic stability and CYP inhibition of our combinatorial libraries is discussed.

**Novel homogeneous FRET-based assay for endopeptidase inhibitors: assay development, steady-state kinetics, high-throughput screening and miniaturization**

*Joe Bradley, Pfizer Global R&D, Sandwich, UK*
*Co-authors: Chris Chambers, Helen Boyd, Emma Faure, Joe Bradley, Simon Dales, Neil Benson, Wilma Keighley and Andreas Sewing*

The growth in compound numbers and subsequent need for increases in throughput and reduction in cost are key drivers of developments in the field of high-throughput screening. Increasingly, there is a move towards fluorescence-based assay technologies, which are ideally suited for screening because they are versatile, homogeneous and amenable to both automation and miniaturization.

We developed a novel FRET-based assay to identify inhibitors of a metallo-endopepidase. This assay uses a novel substrate with a low $K_m$, thus making it cost-effective for high-throughput screening. We have successfully transferred and validated this assay onto our Robolab linear track-screening platform. A full HTS campaign was subsequently conducted in 384-well format, achieving a maximum throughput of 75 000 data points within 24 h.

This poster will illustrate the HTS process from assay development to high-throughput screening using state-of-the-art technology.

**Accelerating knowledge transfer for improved lead-candidate selection**

*John Helfrich, NuGenesis Technologies Corporation, Westborough, MA, USA*

Managing and tracking data through the discovery process requires a compilation of many different types of analytical, biological and image output. This includes the collection, storage and management of relevant scientific information about lead candidates, as well as immediate access to this information for complete compound document creation. Data collaboration across the discovery arena, even across the entire enterprise, is critical to making crucial go/no go decisions about subsequent lead candidate development.

The NuGenesis® Scientific Data Management System (SDMS) allows discovery scientists electronically to view, share, reuse and access data within the laboratory, as well as throughout the enterprise. You can easily capture laboratory and report data produced in any Windows-based software application. These data can be catalogued automatically and accurately for easy retrieval. Immediate access to this electronic information is possible from anywhere around the world using Web technology. This presentation will focus on the principles of good data management that allow for pharmaceutical and biotech discovery facilities to leverage data as an asset, protect valuable intellectual property and ensure the accurate and easy creation of complete compound documents throughout the drug-discovery and development process.

**Elutri-Zone MS: a new SPE system for rapid sample processing and LC/MS analysis**

*John Janiszewski, Pfizer Global R&D, Groton, CT, USA*
*Co-authors: R. M. Olech, R. A. Pranis, J. R. Jacobson, C. A. Perman, B. A. Boman, J. Soldo, R. Speziale, T. W. Astle, M. J. Cole, J. S. Janiszewski and K. W. Whalen*

To increase sample throughput, many recent LC/MS methods have described techniques to shorten and/or simplify the chromatography step(s) before MS analysis. We describe a discrete solid-phase extraction step using Empore SPE membrane to capture the analyte, followed by an elution step that features the rapid quantitative transfer of the elutri zone (pure zone) into the MS. This application was designed primarily for bioanalytical support of high-throughput ADME screening.

An SPE Card having the outer dimensions of a microtitre plate was developed by molding a plastic frame around a sandwich of 3M Empore sorbent (0.5 mm thickness, 8 mm particles) and microfibre support material. Ninety-six discrete elution zones (7 mm diameter, 9 mm centres) were welded into the sheet. The SPE Card was designed to fit in a modified cell harvester (Tomtec) that facilitates SPE processing. The harvester performs sorbent activation, load and wash steps. Once the analyte has been retained, an interference wash is completed and the plate transferred to an elution device (Elutrix, Tomtec). Each well was eluted directly into the MS using flow rates ranging from 1.0 to 3.0 ml min$^{-1}$. A single HPLC pump delivered eluent composed of 5 mM Ammonium Acetate combined with 50–90% methanol or acetonitrile.

The SPE Card and Harvester protocol allow rapid (2–5 min) offline sample clean-up followed by direct elution into the MS detector. This protocol results in the concentration of the sample and reduces liquid-handling steps as dry plates are transferred directly to the Elutrix for LC/MS processing. Owing to the small particle size of the Empore sorbent, the SPE Card can mimic the performance of discrete HPLC columns, similar to those used in high-throughput sample analysis or in online clean-up protocols. Relevant parameters and preliminary results will be described here.

**Combined gravimetric and colorimetric method for the calibration and performance testing of liquid-handling workstations in a GLP laboratory**

*John R. Alianti, GlaxoSmithKline, Research Triangle Park, NC, USA*
*Co-author: Glenn Smith*

Good laboratory practices (GLPs) require that appropriate calibration and performance testing be conducted on all pipetting devices, including automated liquid-handling systems. Routine evaluation of the equipment must be performed to assess both the accuracy and reproducibility (precision) of liquid transfers. Additionally, the testing methods and acceptance criteria must adhere to established company-wide policies (SOPs). These methods must be sensitive as well as being robust and practical. Likewise, the data generated must be easily analysed, interpreted and documented.

Pipetting accuracy is determined by weighing 96-well plates (in replicates of three) before and after dispensing a specified volume of liquid. System accuracy at various volumes is determined and the results used to calibrate the workstation as appropriate (e.g. Zymark RapidPlate Syringe Calibration Factors, Tecan GENESIS Liquid Classes, Packard MultiPROBE II Performance Files). The between-tip precision, individual-tip precision and individual-tip accuracies are all ascertained by subsequent colorimetric testing using a photometric microplate reader. A preformatted Microsoft Excel spreadsheet containing embedded formulas automatically analyses the data and generates a report.

The procedure developed combines the simplicity of gravimetric measurement with the speed of colorimetric microplate reading. The process is generic and can be used to assess or compare the accuracy and precision of any workstation. Automated data analysis and standardized report generation facilitate the process and ensure regulatory compliance.

### Implementing automated sample preparation for GLP and non-GLP bioanalysis to increase productivity in a contract research organization

*John R. Kagel, Charles River Discovery and Development Services, Worcester, MA, USA*
*Co-authors: Larry E. Elvebak, Brian E. Lilley, Jakal M. Amin and James A. Jersey*

Client services for analysis and bioanalysis must: provide reliable results, meet increasingly aggressive timelines, meet increasingly lower unit costs and be performed under appropriate regulatory conditions. Automation was used successfully to satisfy these criteria regarding sample preparation for high-throughput LC/MS/MS GLP and non-GLP bioanalysis in a CRO. The first phase in automating sample preparation involved processing samples in a 96-well format using 96-tip parallel pipetting workstations (e.g. Tomtec) in an open-access environment. The impact of this implementation was to increase productivity by at least twofold. The second phase in automating sample preparation used a liquid handler (e.g. Tecan Genesis) for the preparation of calibration standards and automated reformatting and dilution of samples from tubes or vials into 96-well plates. In addition, calibration and compliance issues related to automated sample preparation will be discussed.

### Octave system: a new automated instrument for the analysis of molecular interactions

*Robert J. Kaiser, Prolinx, Inc., Bothell, WA, USA*
*Co-authors: Karin A. Hughes, Guisheng Li, Deborah D. Lucas, Kevin P. Lund, Chris Pershing, Douglas A. Spicer, Mark L. Stolowitz, Jean P. Wiley, Steve Bailey, Michael Baum, Erik Engstrom, Scott MacInnes, Rich Ward, Craig Yamamoto, George de la Torre, Lynn Hilt, Charles J. Hutchings, Arturo Kozel, Terry A. Weeks, Dwight Bartholomew, Rick Carr, Keren Deng, Jerry Elkind, Tom Martin and John Quinn*

Surface plasmon resonance (SPR) technology is a powerful, label-free method for the analysis of molecular interactions. Prolinx®, Inc., has developed a new automated instrument, the Octave Molecular Interaction Analysis System, that allows this powerful analytical platform to become ubiquitous in life-science research and drug-discovery laboratories. This technological breakthrough is the result of combining the miniaturized Texas Instruments Spreeta™ 2000 SPR sensor with the Prolinx Versalinx™ Chemical Affinity Tools. These technologies have made possible the development of an instrument of moderate cost that incorporates eight independent sensors operating in parallel. The sensor surfaces can be readily and efficiently modified with molecular targets, and exhibit low non-specific binding. Samples are introduced to the sensors from standard microwell plates using an integrated liquid-handling robot. This new instrument will significantly increase the throughput of SPR-based molecular interaction analysis in basic biological science and drug-discovery applications.

### Increased throughput efficiency with dual-column technology in LC/MS/MS sample analysis

*Kathryn B. O'Mara, GlaxoSmithKline, Research Triangle Park, NC, USA*
*Co-authors: Lisa St John-Williams and John A. Dunn*

Recent advances in automation have significantly reduced the amount of time it takes to prepare clinical samples for LC/MS/MS analysis. As a result, the efficiency bottleneck has shifted from sample preparation time to analytical run time on the mass spectrometer. To reduce the mass spectrometry analysis time, the use of dual-column technology was evaluated for three single-column validated LC/MS/MS methods.

For each of the three methods, the HPLC system was enhanced with switching valves and the appropriate programming to allow the unit to function in the dual-column mode. A semi-automated liquid handler was used to perform either protein precipitation or solid-phase extraction using 96-well technology. A triple quadrupole mass spectrometers was used to acquire data using multiple reaction monitoring.

The three quantitative LC/MS/MS methods using dual-column technology are currently being used in a high-throughput laboratory. Each method has been validated to demonstrate acceptable accuracy and precision. Robustness has also been demonstrated by the analysis of thousands of samples in a GLP environment. For each method, the analytical run time has been reduced by

50% and instrument efficiency and sample throughput have been increased twofold.

### Rapid, efficient method for determining metabolic stability

*Kelly Johnson, Waters Corporation, Milford, MA, USA*
*Co-authors: John Erve, Andre Dandeneau and Beverly Kenney*

Modern drug discovery has been transformed by the automation of research. The resulting explosion of data in the discovery pipeline, combined with the pressure to reduce costs and speed up drug-discovery cycles, provides a strong demand for fast and selective analytical methods to produce quality data.

*In vitro* metabolic stability assays provide a rapid estimation of new chemical entities in the discovery phase of pharmaceutical development. These multistep assays routinely incorporate robotic liquid-handling systems to automate the incubation step of test compounds with human liver microsomes in 96-well plates. Metabolic stability is subsequently measured by LC/MS as the amount of substrate metabolized (expressed as the percentage remaining of the initial substrate). The resulting information is crucial in the selection process in determining a compound's potential 'drugability'.

Given the large number of samples generated by the liquid-handling systems in metabolic stability assays, employing automation and higher throughput in every step of the assay has become a necessity. This presentation will demonstrate the use of high-throughput LC/MS methods to analyse samples produced in a metabolic stability assay. We will develop LC/MS methods that significantly increase overall throughput without sacrificing data quality. By incorporating such factors as parallel sample processing, high sample capacity, alternating column regeneration and smaller diameter columns to reduce cycle times, we can analyse the large number of samples produced and ultimately help expedite the drug-discovery process.

### Filter-immobilized artificial membrane (filter-IAM) permeability assay: a new high-throughput *in vitro* drug-absorption model

*Konstantin Tsinman, pION, Inc., Woburn, MA, USA*
*Co-authors: A. Avdeef, D. Voloboy, M. Strafford and B. Kenney*

The assessment of passive transport properties of over 20 drug and natural product molecules was made using the *in vitro* absorption model based on filter-immobilized artificial membranes (filter-IAM), assembled from phosphatidylcholine in dodecane, in buffer solutions at pH 7.4.

Several of the compounds were lactones extracted from the roots of the kava-kava plant. Experiments were designed to test the effects of stirring during assays and the effects of varying the assay times.

The highly mobile kava lactones permeated in the order dihydromethisticin > yangonin > kavain > methisticin > desmethoxyyangonin. Other molecules in the study ranked: phenazopyridine > testosterone > propranolol > ketoconazole > piroxicam > caffeine > metoprolol (proposed BCS internal standard) > terbutaline.

Stirring during assay significantly increased the observed permeabilities for highly mobile molecules.

In addition to permeability measurements, membrane retention of compounds was determined. Yangonin, desmethoxyyangonin, ketoconazole and phenazopyridine were > 60% retained by the artificial membranes containing phospholipids.

The influence of hydrogen bonding was explored by determining permeabilities using filters coated with dodecane free of phospholipids. The membrane transport of phenazopyridine (strong hydrogen-bond donor) is about twice as fast and retention is about twice reduced in the inert lipid membranes compared with phospholipid-based membranes.

In the filter-IAM method, concentrations were determined by microtitre plate UV (190–500 nm) spectrophotometry and by LC/MS. Higher-throughput was achieved with direct UV by the use of 96-well microtitre plate formats and with LC/MS by the use of cassette dosing (5-in-1).

### Seamless integration of information in a pharmaceutical development environment: integrating technology from LabWare, NuGenesis, VelQuest and Waters

*Kurt Roinestad, Purdue Pharma LP, Ardsley, NY, USA*

This presentation will describe Purdue Pharma's approach to an integrated solution between Laboratory Information Management Systems (LIMS), Chromatography Data Systems (CDS), Process Management and Compliance Systems (PMC), and Scientific Data Management Systems (SDMS). It will describe the vendor-selection process and how Purdue Pharma created an environment to produce a best-of-breed information solution to creating the paperless laboratory. Working towards the creation of a paperless laboratory has the potential to result in significant benefits including resource liberation and an electronic compliance platform for process management and data review.

This paper will describe the process of working with four vendors to create a complementary data management solution to Purdue Pharma's needs. The process of logging in samples, creating work-lists, executing analytical methods, capturing data electronically, storing electronic records and submitting reports will be reviewed. After data creation, electronic data review will be discussed enabling electronic 'instant replay' of laboratory information.

The primary benefits of this complementary solution approach hold the possibilities to do the following.

- Increased capacity by liberating valuable resources from rework loops and the manual data review process.
- Leveraging the approach to validation of the overall solution saving time associated with multiple computer validation plans and execution.

- Promise of reduced time to market for new drugs by using a paperless environment where possible.

This presentation will describe the challenges, successes and experiences surrounding the task of integrating complementary information systems in a pharmaceutical development environment.

### High-throughput robotic workstation for performing gene-expression assays

*Michael A. Kuziora, Gene Logic, Inc., Gaithersburg, MD, USA*

Metabolism and growth are dependent on a highly orchestrated interplay of a variety of proteins within cells. These proteins function as enzymes in metabolic pathways, signalling molecules for communication between and within cells, and as various cellular structural components. In a disease state, cells often modulate the amounts of specific proteins and may produce new proteins not normally found in a particular type of cell.

The alterations in the proteome observed in the diseased state most often result from changes in gene expression levels within a cell. Gene Logic believes that pharmaceutical companies can reduce the time, risk and cost associated with drug discovery if they know the expression levels of genes that play roles in the disease-associated pathways. Such knowledge may help them discover drug targets, screen drug leads, and predict toxic and pharmacological responses to drug leads.

Using the Affymetrix, Inc. GeneChip® micro-array platform, Gene Logic has measured the expression levels of thousands of genes from a diverse range of normal and diseased tissues to create a reference gene expression database called the Gene Express® Suite. The bioinformatic analysis tools incorporated in the Gene Express Suite facilitate the identification of distinct sets of genes whose expression is consistently altered in a particular disease state. The expression patterns of these gene sets become a molecular fingerprint of the disease and thus not only reflect the disease status of metabolic pathways, but also could also serve to indicate the effect of potential treatments when affected cells are exposed to a potential drug.

A primary goal of a pharmaceutical company is to discover drugs that restore the normal functioning of the disease-affected pathways. In one scenario, a pharmaceutical company could use the information in the Gene Express database to identify a small set of genes that indicate a disease state. The expression of this gene set is then monitored when an appropriate cultured cell line that represents a disease state of interest is exposed to chemicals from a compound library. A potential drug can be identified for further characterization if, for example, it is found to restore expression levels of the gene set to normal levels.

Unfortunately, the use of large micro-arrays to measure gene expression of a few genes in a high-throughput screen is not economically feasible at this time. We therefore investigated the use of alternative methodologies to measure gene expression. Bayer Corporation's Quantigene™ assay, which uses branched DNA (b-DNA) for signal amplification, and provides a sensitive and reproducible method for measuring mRNA levels of a small number of genes. The assay's 96-well plate format and simple handling procedures makes it highly amenable to high-throughput robotics. This talk will describe a Zymark Staccato™ Workstation designed to perform the hybridization, amplification and signal-detection steps of the Quantigene assay.

### Multivariate calibration of a UV-VIS fibreoptic probe used for direct measurements in a Zymark XP-based automated dissolution system

*Lars A. Svensson, AstraZeneca, Molnda, Sweden*
*Co-authors: Rimstedt Eva and Svensk-Ankarberg Anna*

Correlation between drug dissolution results obtained by both liquid chromatography (LC) and ultraviolet-visual (UV-VIS) spectrophotometry has been performed by multivariate calibration.

The dissolution of tablets or capsules, stored and exposed to harsh conditions (high humidity/elevated temperatures) at different degrees, was carried out by an automated system based on the Zymark XP robot. The system had the capability of withdrawing samples to be subsequently prepared into LC vials at the same instance as an UV-VIS spectrum was recorded via a fibreoptic probe. The same probe was used in all vessels and could be attached directly adjacent to the filter tip for the sample withdrawal on one of the robotic hands.

The subsequent multivariate calibration was performed with Simca-P 8.0 (Umetrics AB, Sweden). Most of the calibration is explained in two components, with the first mainly corresponding to the UV spectrum and the second taking into consideration the baseline offset.

### High-throughput chemistry: an integration of chemistry, automation and informatics

*Li Chen, Hoffmann-La Roche, Inc., Nutley, NJ, USA*

One of many impacts that combinatorial chemistry has been made in the last decade is to stimulate intellectual creativity to invent new and more efficient ways of making new compounds. Although much effort has been investigated into developing synthetic methods, automated synthesizers and analytical tools, the high productivity of compound library synthesis cannot be achieved without the integration of chemistry, automation and informatics into an efficient process. I will emphasize what we have learned in how to establish an infrastructure that integrates functional modules containing a diverse set of tools for high-throughput organic synthesis applications. This modular approach provides the maximum efficiency of combinatorial tool functions to support multiple project teams for drug lead generation, exploration and optimization.

The agenda includes the following.

- HTOS process design.
- Pro- and post-synthesis automation.
- Synthetic chemistry method.
- HT analysis and purification.
- Data management and chemo-informatics.

**Living Chip™: a nanofluidic platform for ultra-high-throughput, massively parallel synthesis, storage and screening (MPS$^3$)**

*John R. Linton, BioTrove, Inc., Cambridge MA, USA*

The Living Chip™ is a nanotitre plate consisting of a uniform and addressable through-hole array. The though-holes are nominally 300 μm square and 500 μm deep, giving each well a 50-nl volume. Proprietary coatings make the surfaces of the plates hydrophobic and the interior of the wells hydrophilic. This allows the samples to be held in the wells by surface forces and prevents sample contamination from adjacent wells. Ten thousand-well chips are currently in use and 100 000-well chips are in production.

Massively parallel mixing of assay components takes place when chips containing different components are stacked such that the through-holes align. The chips are imaged using a CCD array for readouts such as absorbance, fluorescence and luminescence. Detection is performed in a transmission geometry, taking advantage of the bottomless wells. These key features and the sophisticated robotics built by BioTrove allow for the rapid acquisition of biochemical information, on the order of $10^7$ measurements per day. Combining nanolitre reaction volumes and a simple interface to microtitre plates, the Living Chip™ format conserves compound libraries, increases analytical capabilities and decreases costs. The platform has applications in all aspects of drug discovery from materials' handling and storage, target ID and validation, to lead discovery and optimization.

Cell culture in the Living Chip™ using yeast (*S. cerevisiae*) and bacteria (*E. coli*) shows similar growth characteristics to cells grown in bulk. Additionally, *E. coli* cells expressing GFP inoculated into isolated channels show no crosstalk.

Diverse biological libraries can be introduced to the chip using a simple dip loading procedure. This technique was used by BioTrove collaborator Genofocus to dip load cells expressing a lipase enzyme library into a Living Chip™. The cells were grown up overnight and then stacked with a chip containing a tagged substrate so that wells containing clones with increased lipase activity yielded a greater fluorescence signal. The 'hits' were harvested by a puff of air from a microsolenoid valve positioned above the well, into a microtitre plate waiting

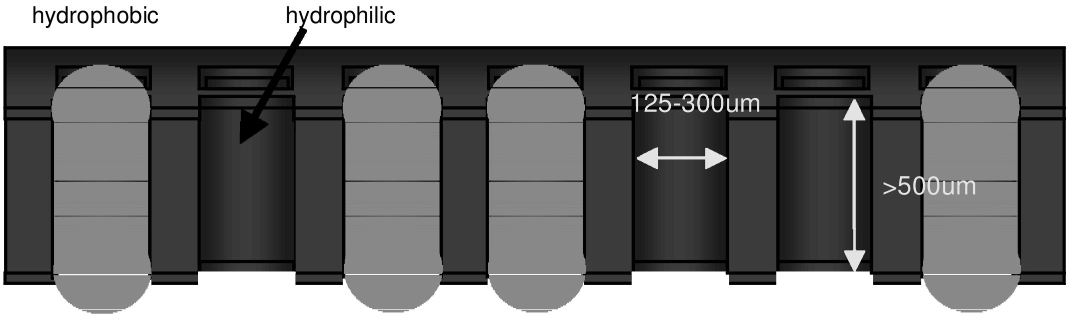

*Figure 1. The Living Chip™: a nanotiter plate. The surfaces are hydrophobically coated and the wells are hydrophilic to contain and isolate samples.*

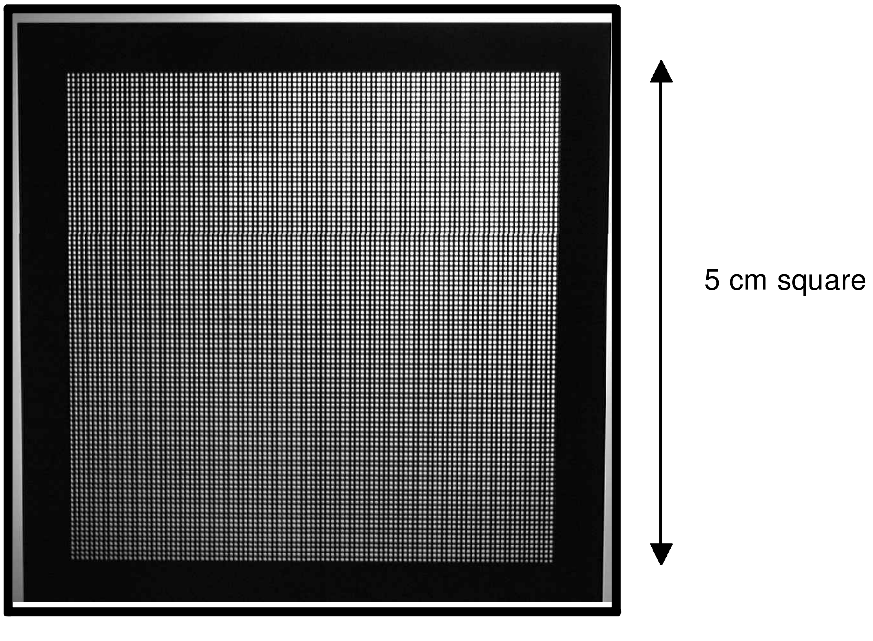

*Figure 2. 10 000 well chips (above) are currently in use and 100 000 well chips are in production.*

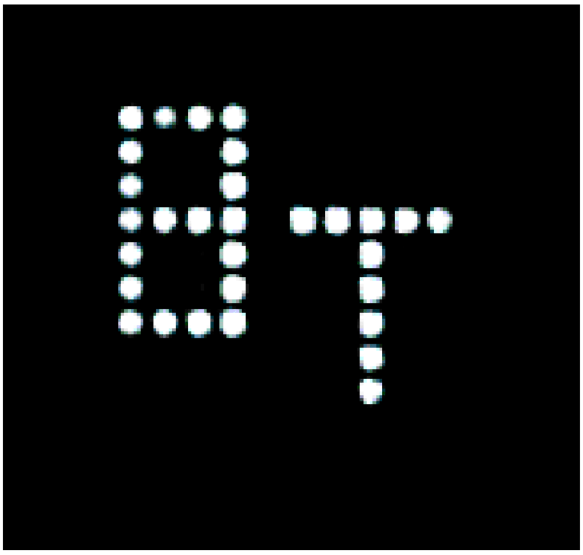

*Figure 3. Isolated wells: all wells loaded with media. Cell expressing GFP innoculated into wells show no crosstalk after culturing.*

below. Kinetic analysis confirmed the discovery of a lipase mutant with a 20-fold higher activity relative to the wild-type. This screening consisted of 30 000 assays performed by one person in less than 2 days, and would have taken several scientists weeks using conventional means.

### Next step in miniaturization: submicrolitre assays in 96-well formats

*M. J. Wildey, R. W. Johnson Pharmaceutical Research Institute, Raritan, NJ, USA*
*Co-author: C. Fleming*

Some of the challenges and goals in many of today's screening laboratories are to investigate, evaluate and validate new techniques that will enable the reduction in cost, provide an increase in efficiency and strengthen the quality of screening data. Towards the end of reducing cost, we have been a Beta test site for a novel 96-well submicrolitre assay system called Arteas. The architecture of Arteas offers a solution to the problem of evaporation in small-volume assays.

We have successfully performed a fluorescent-based enzyme assay in Arteas with a reaction volume of 400 nl. We successfully reproduced published control $IC_{50}$s and routinely generate Z'-factors in the range 0.6–0.7. We will show these data and variability around some tests performed with actual library compounds. Translation of this device to production screening will be dependent upon the successful integration of nanoliquid handling onto our robotic platforms. Initially, we will integrate this new liquid-handling option onto our Fast Track robotic platform. Progress on this translation will be addressed.

### Automated compound dilution and presentation for determination of $IC_{50}$ data

*Malcolm Willson, Systems Research, GlaxoSmithKline, Stevenage, UK*
*Co-authors: David Brown and David Hayes*

Programme-targeted SAR screening within Systems Research at Stevenage calls for a process able to progress compound activity determination (80 to $> 320$ compounds/week) in real time, enabling ongoing chemistry to continue based on known biological activity.

Efficient compound dilution and generation of assay plates is required to meet weekly turn around times for $IC_{50}$ and cross-screening data generation.

Comparison of manual across-plate dilution, vertical dilution down a series of plates and Z-dilutions within a series of 384-well plates will be discussed.

Use of the twin head Biomek FX uses the flexibility of both the 96- and 384-tip heads, allowing Z-dilutions, a very efficient method for automated generation of $IC_{50}$s. By having space on the stock plates for several extra compounds, a selected range of 'standards' covering several assays can be included to enable assay performance and automation QC to be monitored for each set of compounds, along with sufficient control/blanks for statistical analysis of assay performance.

Cloning of assay templates reduces IT resource requirements for data analysis as templates can be cloned rather than written from scratch.

If compound numbers increase, the system can be converted to run vertical dilutions down a series of plates (different dilution per plate). However, this only works if the assays are stable enough for the increased length of assay due to the higher plate numbers.

Conversion to 1536 plates doing Z-dilutions from 384-well stock plates is also an option that would be possible when liquid-handling devices can cope with smaller volumes for assays.

### Lead generation and optimization: integrated data mining and informatics in drug discovery

*Charles J. Manly, Discovery Technologies, Neurogen Corporation, Branford, CT, USA*

Drug discovery today includes considerable focus of laboratory automation and other resources on high-throughput technologies, but lead generation and optimization to clinical candidates continues to be a lengthy and costly process. The real benefit of today's technologies is beyond the exploitation of each individually. Only recently have significant efforts focused on effectively integrating these complex discovery disciplines to realize their larger potential. Informatics, computational chemistry, virtual screening and data mining play a large role in this integration and in increasing the efficiency of the drug-discovery process.

### Survival skills for managing robotics and automation: how to outwit, outplay, outlast

*Maria DeGuzman, Affymetrix, Inc., Santa Clara, CA, USA*

Our experience in automating a genomics' laboratory for single nucleotide polymorphism (SNP) detection has shown us that automation projects are not often completed in the planned or expected manner. With automation playing an ever-increasing role in the research and development laboratory, we will discuss why some projects succeed while others fail. For instance, what are the real bottlenecks to address? Does experience make a difference? Is management expectation realistic? What are the advantages and disadvantages of custom or commercial automation? What are the roles of the scientists, engineers, and laboratory managers in such projects?

The recent completion of our high-throughput screening project has enabled us to look back objectively and answer these questions for our genomics' application.

The high-throughput screening project at Affymetrix began in 1999. The initial goals of the project were to screen for SNPs across 40 unrelated individuals in an automated fashion. In the initial stages of this project, the process), which encompassed sample preparation to scanning, was done manually. This provided an output of about 2.3 Mb of sequence a week for every 4.5 people. With the collaborative effort of the executive management group, the high-throughput screening laboratory, the engineering group and the bioinformatics group, we set out to accomplish this task.

Unfortunately, as the project continued, conflicts and difficulties arose. Each group had its own expectations for the project that were not necessarily communicated with the others effectively. This led to many problems including delays, miscommunications, unrealistic expectations, frustration and blame being placed on one another. In addition, another facet compounded the already difficult process: reality. We found several instances where reality interfered in the development of the project including budget, time, resources and process bottlenecks. We also saw evidence of conflicts such as when we needed to decide between 96- or 384-well plates, tubes or plates, custom or commercial or semi-automation, or even conflicts between the scientists and the engineers.

Fortunately, we realized many of these issues and managed them effectively. We also proceeded to perfect the process in other ways using protocol enhancements and microscaling improvements. This increased our throughput to 1.4 Mb per day for every two people while reducing reagent costs.

At the conclusion of this project, approximately 25 000 genes were screened covering 8.2 Mb of the genome (including genes, regulatory regions and STSs). Since most of the projects were tested against 40 individuals, we screened 24 million bases of dsDNA and identified 15 682 SNPs that have been deposited to the SNP public database. In addition, much of the software, automation and protocols developed during this project have been incorporated into other internal research laboratories.

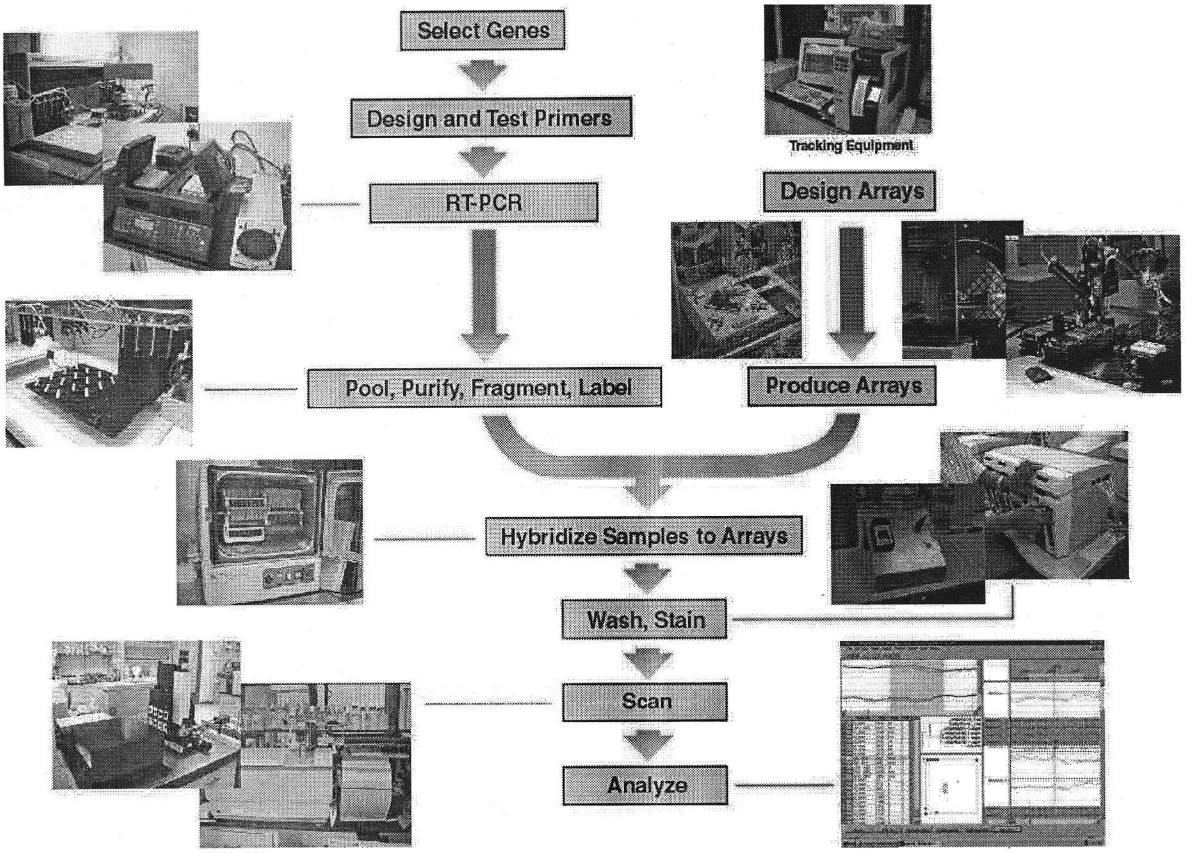

### Stand alone extractor for semi-automated method development and validation

*Maria Styslo-Zalasik, R. W. Johnson Pharmaceutical Research Institute, Raritan, NJ, USA*
*Co-author: Kathleen Cirillo-Penn*

In the past 10 years, laboratory automation has become increasingly important for routine chemical analysis testing in the pharmaceutical industry. The use of automatization or semi-automated laboratory equipment can reduce the laboratory flow-through time of the samples in the QC area. Likewise, automated and semi-automated equipment can be used as a tool for research during method development and validation.

This presentation will illustrate how semi-automated sample preparation can afford rapid optimization of method parameters during early-stage method development and validation. The potential dissolution rate and solubility issues with high sample concentrations can be avoided through the evaluation of data from parameter optimization of the sample preparation using semi-automated instrumentation. Examples for the use of a stand alone extractor in an assay/purity method validation will be presented.

### New automated instrument for the characterization of biomolecular interactions

*Mark L. Stolowitz, Prolinx, Inc., Bothell, WA, USA*

Surface plasmon-resonance technology is a powerful, label-free method for the analysis of biomolecular interactions. However, the use of this technology is limited by the cost, throughput and complexity of existing instrumentation and chemistries. Prolinx has developed a new instrument that will address these limitations and allow this powerful analytical platform to become ubiquitous in life science research and drug-discovery laboratories.

This technological breakthrough is the result of combining the Texas Instruments Spreeta® 2000 chip with Prolinx's Versalinx™ Chemical Affinity Tools. These technologies enabled the development of an instrument of moderate cost that incorporates eight parallel sensor surfaces that can be efficiently modified with molecular targets and exhibit low non-specific binding. The presentation will encompass technology, design and applications of this new instrument.

### Automation of an infectivity assay for the quantitative analysis of virus in biopharmaceutical products

*Mervyn Cadette, GlaxoSmithKline, Beckenham, UK*

Relative to chemical assays in a pharmaceutical development environment, biological assays are relatively 'low throughput' in comparison. However, the complexity of assays associated with biological products is often of a different magnitude. Biological assays become attractive automation candidates based upon their complexity coupled with high demand for their use in biopharmaceutical analysis.

The Tissue Culture Infectious Dose 50% (TCID50) assay was used for the quantitation of viral titres used in vaccine products.

These assays required sterility, therefore requiring all equipment to be housed in Class II microbiological safety cabinets. The TCID50 assay was programmed on a Tecan RSP 200 using eight disposable-tip liquid-handling probes in conjunction with a robotic manipulator arm. Culturing of plates containing tissue culture, to support the TCID50 assay, was automated using a Zymark Twister coupled to a Multidrop dispenser.

Scheduling and liquid-handling software were combined to execute this automated assay. The use of the 'joblist' function allowed the operator to select from a database of predefined dilutions within the Logic pipetting software.

A formal comparison of the automated TCID50 assay against the manual TCID50 was performed and proved comparability.

### Integrated approach to high-throughput sample processing, characterization and purification

*Michael L. Moore, GlaxoSmithKline, King of Prussia, PA, USA*

The extension of high-throughput synthesis in support of lead optimization has imposed increasingly stringent requirements on compound characterization, purity and accurate concentration determination. We developed an integrated and highly efficient process for compound analysis, purification and sample processing with a capacity of 100 000 compounds/year at a 20-mg scale. Purification is driven by ultrahigh-throughput analytical LC/MS, which minimizes the number of fractions collected and analysed. Custom software with a browser-based front end is employed to track samples and provide the required data to robotic workstations.

### Managing laboratory automation in the postgenomic era

*Michael R. Kozlowski, Axiom Biosciences, San Diego, CA, USA*

The completion of the cloning of the human genome has provided the drug-discovery community with a wealth of potential drug targets. At the same time, it has produced challenges to the way in which drug discovery is done. Formerly, the mandate of drug discovery was to screen a relatively small number of well-validated targets against an immense number of compounds. Now, drug-discovery scientists are faced with processing a very large number of minimally validated targets. Most targets were formerly single proteins, which are amenable to screening in highly reductionist systems. Now it is clear that most biological processes, including those contributing to pathological states, must be the result of complex interactions between proteins. A way must be found to address this new level of complexity. In addition to these challenges, the availability of the entire sequence of the human genome raises expectations for the rapid introduction of more, and better, drugs.

These challenges demand new ways of thinking about how we carry out drug discovery, and about drug-discovery automation, from assay development to ADMET profiling. This talk will discuss some of these new approaches.

### High-throughput screening inhibition assays to evaluate the interaction of Pfizer proprietary compounds with cytochromes P450

*Michael West, Pfizer Global R&D, Groton, CT, USA*

*Co-authors: Larry Cohen, Alfin Vaz and Shawn Harriman*

A fluorescence-based drug interaction assay using recombinant CYPs and a cocktail of CYP-specific probes in human liver microsomes was assessed as higher throughput methods for evaluating the potential for inhibition of CYP1A2-, CYP2C9-, CYP2C19-, CYP2D6- and CYP3A4-mediated metabolism. Comparisons of $IC_{50}$ obtained with the fluorogenic and conventional drug probes in recombinant CYPs were similar for CYP1A2, 2C9, 2C19 and 2D6, but not for CYP3A4.

Additionally, using a single-point estimated $IC_{50}$ approach, compounds that were shown to be inhibitors using conventional drug probes with human liver microsomes were also classified as inhibitors in recombinant CYPs using the fluorescent probes. For the cocktail approach, it was shown that the CYP-specific reactions were not altered in the presence of multiple probes as indicated by no distinguishable effect on $K_m$ or $V_{max}$. As expected from this result, the $IC_{50}$s generated in the cocktail incubations were in good agreement to those obtained from individual incubations.

### GigaMatrix™ ultrahigh-throughput screening platform

*Mike Lafferty, Diversa, San Diego, CA, USA*

The myriad of microbes inhabiting this planet represent a tremendous repository of biomolecules for pharmaceutical, agricultural, industrial and chemical applications. Diversa Corporation has the unique capability of accessing this microbial diversity by taking a culture-independent, recombinant approach to the discovery of novel proteins and small molecules. Diversa's discovery programme uses genes and gene pathways captured from nucleic acids extracted directly from the environment, which are then constructed into complex, 109-member environmental libraries. These libraries often contain up to 5000 different microbial genomes and, thus, require high-throughput screening methods to cover their diversity effectively.

Diversa has developed GigaMatrix™, a new ultrahigh-throughput screening platform. GigaMatrix plates have > 100 000 bottomless wells in the same footprint as a microtitre plate. The platform is automated and capable of screening 1 billion clones per day. Less equipment time and manpower are required and assay costs are dramatically reduced as compared with traditional microtitre plate-based screening. The power of the GigaMatrix platform to discover novel enzymes, small molecules, protein therapeutics and other bioactive molecules will be presented.

### Building an electronic data-handling, compliant, foundation for the QA analytical laboratories at Westborough

*Mike Stroz, AstraZeneca, Westboro, MA, USA*

A significant portion of QA laboratory activities are dedicated to compliance and data handling in a paper-based system. AZ Westborough is implementing an electronic environment in the laboratories to ensure 21 CFR Part 11 compliance, improve efficiency and reduce costs. The presentation will discuss the applications selected, and the validation and system architecture being installed to achieve these goals.

### Online SPE by column switching: another form of bioanalytical laboratory automation

*Min Chang, Abbott Labs, Abbott Park, IL, USA*
*Co-authors: Huong Mai, Anita Shen, Brendan Swaine, Qin Ji and Tawakol El-Shourbagy*

Time event-controlled column switching valves have been available to analytical scientists since the mid-1970s. Earlier uses of the column-switching valve included removing late elution peaks from the HPLC run, autosampler sharing, two-dimensional HPLC separation (heart cut), inline filter/guard column regeneration, online concentration, unattended column selection and fractions' collection. Although there is at least one column-switching valve in an HPLC system (the injector valve), the idea of the addition of another valve has not been widely accepted. Analytical scientists have turned away from the technology possibly due to the lack of ruggedness of the first-generation air-actuated valve and other additional pieces of HPLC equipment including columns.

Recently, with the availability of commercial online solid-phase extraction system(s), the column-switching technique has become an acceptable option for bioanalytical sample preparation. At Abbott Laboratory, we have developed several online solid-phase extraction HPLC methods using automated valves, an internal reversed-phase guard cartridge and HPLC equipment by Shimadzu and Agilent. Internal standard fortified plasma were clarified by either centrifugation or filtered before the HPLC injection. These methods have been used successfully to analyse two Abbott compounds and their metabolites. This application of automated column-switching valves has provided an alternative to offline solid-phase extraction and liquid–liquid extraction and made it possible to select the best technology to increase assay throughput and sensitivity.

### Automation and compliance in quality control laboratories

*Muhammad Albarakeh, Barr Laboratories, Inc., Pomona, NY, USA*
*Co-authors: Richard Ashley and Timothy Breuninger*

Current laboratory demands related to the high throughput of samples, the need to keep costs and expenses minimal, yet keep the release function flowing to prevent product backorder, have necessitated the use of laboratory automation and robotics systems. As a result of this automation, huge amounts of samples and related data are processed and generated.

Without a properly controlled and validated system, the very tools implemented to process high amounts of samples can bring your release function to a halt. Overviews on how to prevent cGMP logs jam and reach a 'zero backorder' release of product will be presented.

### Methods' development for monoclonal antibody screening: how to think like a robot

*Nanci E. Donacki, MedImmune, Inc., Gaithersburg, MD, USA*

A single fusion for the development of monoclonal antibodies will often generated 20 or more microplates that need to be screened. ELISA (enzyme-linked immunosorbent assay) is the most common method for screening newly developed monoclonal antibodies for antibody production and specificity. The method, although specific for the antibodies, is highly repetitive for each step and can easily be automated. However, the way the assay is performed at the benchtop is not always the best way for an automated system to run the assay. Tips and techniques for transferring an assay from benchtop to automation will be presented.

### Lean manufacturing and six sigma: an evaluation of the impact of these concepts on laboratory automation

*Nigel North, Pharmaceutical Development, GlaxoSmithKline, Ware, UK*

The concepts of lean manufacturing and six sigma are now beginning to be applied to pharmaceutical manufacturing processes. Lean manufacturing has been successfully applied for many years in the automotive and aerospace industries with the key goal of reducing waste. Six sigma is a more recent concept involving reducing variation in manufacturing processes which has been implemented in the semiconductor industry resulting in significant cost savings. The combination of lean manufacturing and six sigma provides a powerful combination of principles to deliver robust manufacturing processes with the elimination of non-value-added activities. The effect of these concepts on how we approach automation in the laboratory will be examined together with providing some perspectives on new technology that will be required to meet these challenges.

### Applications of the Zymark Staccato for *in vitro* drug metabolism studies

*Heather Sulkowski, Boehringer Ingelheim Pharmaceuticals, Inc., Ridgefield, CT, USA*

*Co-authors: J. Richard Mountfield, Donald Tweedie and Drané O'Brien*

Advances in genomics, combinatorial chemistry and pharmacology screening have led to new challenges within drug metabolism due to large numbers of compounds requiring *in vitro* evaluation. To meet these demands within Boehringer Ingelheim Pharmaceuticals, Inc., the Zymark Staccato was evaluated as a robotic tool for conducting multiple *in vitro* assays. The criteria for an automated system included the following.

- A 96-well head allowing for maximum sample throughput.
- Thermal block to maintain constant temperature.
- Flexible deck layout.
- Open-access capability

As part of the evaluation process, assays were compared using both manual and automated methods. The results are described in this presentation

Automation of several key *in vitro* metabolism assays has been achieved. The results indicate that the automated assays allow for a higher throughput while maintaining a high degree of precision. The work will be extended to additional *in vitro* assays, and the strategy of comparing manual versus automated processes will be continued as part of the validation procedures.

### Analytical method validation: an automated software approach

*Patricia A. Fowler, Waters Corporation, Milford, MA, USA*
*Co-author: Michael Swartz*

Method validation is a tedious process performed to determine if an analytical method meets the requirements for its intended purpose. In the regulated laboratory, method validation may take many days to perform the necessary analytical tests. Data reduction and the statistical analysis of results performed can be a very time-consuming process. There is also a greater possibility of introducing error when calculations are performed manually. With the use of automated software to perform these calculations, method validation is much faster and easier, with less chance for error.

In this poster, we will show that an analytical method was validated using automated software. Chromatographic results were directly accessed from a relational database bypassing manual intervention. Statistical calculations were performed automatically and a report generated showing the results of the analyses from the Student, Cochran, Dixon and Fisher tests. Graphs were generated representing the results of the statistical analysis. In addition, we will show that the data reduction and statistical calculations necessary to validate the method, complete with the necessary documentation and report generation, are completed in significantly less time.

### Streamlining the dissolution test: automated HPLC techniques

*Patricia A. Fowler, Waters Corporation, Milford, MA, USA*
*Co-authors: Kelly A. Johnson, Michael E. Swartz and Charles H. Frasier*

The fast pace of the pharmaceutical industry requires laboratories to reduce the analytical burden of their test procedures and increase productivity while still satisfying regulatory compliance. There are several ways - to meet these challenges in the dissolution laboratory. By automating the dissolution process, pharmaceutical laboratories eliminate the slight variations that may occur in manual methods, insuring reproducible data, higher throughput and cost reduction. Validated single-source software control of the entire system, as well as dissolution data acquisition, calculations and reporting, can further streamline the work flow while maintaining FDA compliance with 21 CFR Part 11.

Automated online HPLC dissolution systems can pool dissolution samples significantly to save time. Similarly, automated sampling at shorter intervals and analysis of a large number of samples by online HPLC may provide a more complete solution for the decision-making process in the early stages of drug development. In addition, these systems must be capable of handling increasingly complex formulations such as multiple actives and widely varying dosage levels, as well as different media types such as buffers and surfactants.

Our poster shows applications used to help reduce the analytical burden and increase the sample throughput and productivity in the laboratory.

### Homogeneous high-throughput SNP assay direct from genomic DNA

*Patrick B. Cahill, Genome Therapeutics Corporation, Waltham, MA, USA*
*Co-authors: Dina Weymouth, Cori Gustafson, James Hurley, Michele Bakis, Veena Kamath, Doug Smith and Lynn Doucette-Stamm*

Genome Therapeutics Corp has developed a high-throughput SNP assay based on the ability of some DNA polymerases to proofread the sequence as they extend. This Exo-Proofreading SNP assay can be accomplished, from sample addition to detection, in one tube. In addition, this SNP assay has the capability to be performed directly on genomic DNA. Results will be presented for this assay on multiple SNPs screened on large populations. The results will be compared with ASO and RFLP data with discrepancies resolved by sequencing. This assay provides a new, highly robust and fast method for large-scale SNP screening required for human disease and pharmacogenetic studies.

### Development of a lead identification platform for kinase drug discovery

*Paul Gallant, Millennium Pharmaceuticals, Inc., Cambridge, MA, USA*

The goal of any drug-discovery process is to eliminate quickly 'poor' leads while rapidly advancing those with the highest potential. Millennium has used a combination of novel and mature technologies to establish a system to progress novel kinase targets rapidly through assay development, high-throughput screening, hit validation and hit characterization. By selecting focused technologies, automation, secondary screens and IT, an integrated process has been constructed which is capable of handling multiple targets quickly while identifying the compounds with the highest potential for lead optimization. This presentation will discuss the platforms chosen for assay development, high-throughput screening, hit confirmation and potency/selectivity determinations. A process will be described to show how using a standard well-defined system can effectively move multiple kinase targets rapidly into the 'Lead Optimization' stage.

### Optimization and automation of bioanalytical methods

*Peter D. Bryan, Forest Laboratories, Abbott Park, IL, USA*
*Co-authors: Min S. Chang and Anita Shen*

Now that LC-MS has become the technique of choice in the GLP bioanalytical laboratory, the workflow bottleneck has shifted from the separation–detection to the sample-processing portion of the bioanalytical method. To address the sample-processing bottleneck, efforts have been concentrated on the conversion and validation of existing liquid–liquid extraction (LLE) to solid-phase extraction (SPE) methods. Profound increases in sample throughput have been achieved using LC-MS with automated SPE in the 96-well format over manual LLE extraction HPLC methods. Validation of automated 96-well format SPE methods have shown their equivalence to manual LLE extraction. Automated 96-well format SPE is also much less tedious and allows the analyst to concentrate more on data analysis and compliance issues. For routine analysis, transfer from clinical tubes to the 96-well format has been accomplished using either the Biomek 2000 or the Hamilton MicroLab AT. Automated SPE is then performed on the Biomek 2000.

Additionally, semi-automated method development for 96-well format SPE has been automated using the Biomek 2000.

### How small is enough?

*Peter Grandsard, Amgen, Inc., Thousand Oaks, CA, USA*
*Co-authors: Jim Petersen, Brian Rasnow, Mike Johnson, Chuck Li, Les Walling and Doug Overland*

A significant technical and organizational challenge facing the biopharmaceutical industry is the incorporation of miniaturization technologies into their business. The successful application of these technologies requires answers to the following questions. What is this new miniaturization technology? Which technologies should be applied to which workflows? Why? When should they be implemented? Should a technology be implemented early, through some sort of technology access programme (TAP), or should one wait until it becomes commercially available? Our views on these matters will be presented.

In collaboration with other Amgen R&D groups, the Research & Automation Technologies department has been actively cultivating critical collaborators with knowledge of submicrolitre liquid-handling, micromachining and detection technologies. For example, our TAP with Caliper, Inc., has increased our institutional understanding of miniaturization applications in the drug-discovery process flow, most particularly in small molecule screening and DNA analysis.

Miniaturization at Amgen has invoked a cascade of new projects or at least studies. The goals of these projects are to find solutions for interfacing any miniaturized platform to the 'macro-world', such that the advantages of miniaturization are not undone by integration or certain interfaces. Operations that need to undergo changes to benefit fully from miniaturization include the storage and retrieval of small molecules, detection and compound handling during screening, and information management. We will discuss some of our findings and solutions.

### Automation and tracking of combinatorial libraries

*Phil Small, Tripos Receptor Research, Bude, UK*

A process-integrating design, synthesis and analysis of combinatorial libraries has been implemented at Tripos Receptor Research. Crucial to the success of this process has been the development of a proprietary informatics system. This has so far been developed to manage reagent inventory, track samples, and record data from synthesis and analysis to provide a valuable database of compound information.

The combination of Tripos's proprietary design software with automated synthesis and informatics leads to the production of drug-like libraries with well-defined purity and full synthetic history.

This presentation will cover what were considered the important aspects involved in setting up and managing an automated chemistry facility.

### Automating pH solubility and stability determinations conducted in support of early development

*Phil Waters, GlaxoSmithKline, Research Triangle Park, NC, USA*
*Co-authors: James Ormand, David Igo and Pingyun Chen*

Evaluation of the equilibrium solubility and chemical stability of compounds as a function of pH aids our understanding of the bioavailability of a drug candidate. Additionally, these data lead to rapid development of formulations for use in preclinical and clinical studies. Conducting these measurements often requires a significant amount of human labour and time. Reducing the labour and time burden is expected to increase the frequency with which these measurements can be applied, thus increasing sample throughput and improving the quality of decisions impacting compound selection and evaluation.

This presentation will describe sample-handling methodologies and automated instrumentation developed to conduct solubility measurements as a function of pH. The use of these methodologies and instrumentation has improved both throughput and efficiency while maintaining the accuracy that can be achieved with manual methods. The accuracy and precision of the system will be illustrated using data on model compounds as well as drug candidates. Finally, we will describe the strategy and process used to prepare samples for stability determinations at user-defined pHs, and how this process is done in concert with the solubility determinations.

### Development of novel micro-array technology for cell-based assays

*Quiyang Zhang, Cytoplex Biosciences, Plano, TX, USA*
*Co-author: Alex Freeman*

Cell-based assays are increasingly used for high-throughput screening in drug-discovery programmes because of their high information content. For the implementation of miniaturized cell assay, micro-arraying is an essential part of the technology. Initially, we had performed cell-adhesion studies and evaluated five different substrates—polystyrene, polycarbonate, silicon, glass and PDMS—and found that with polylysine coating, the glass, silicon and PDMS substrates served well for cell attachment. However, the conventional well-less spotting and assaying method was difficult to implement in producing a predetermined array pattern of cells.

To overcome the above difficulty, we further developed both bottomed and bottomless well arrays to address the need for cell culturing and assays. PDMS and glass were used as the bottom surfaces for both bottomless well silicon substrates and bottomed wells. Novel liquid distribution arrays were fabricated in silicon to facilitate initial coating and liquid exchange from the entire array. The arrays were implemented for culture of adherent and non-adherent cells, immunochemical assays, and automatic liquid transfer all in high-density format. The current array platform will be also useful in cell library construction, combinatorial library synthesis, and continuous homogeneous and heterogeneous assays.

### Adaptive powder-dispensing system

*Rajesh K. Maheshwari, Schering-Plough Research Institute, Union, NJ, USA*
*Co-authors: Annaniy Berenshteyn, Gary Kowalski and Joseph Norgard*

This poster is about a robotic powder dispensing system we have developed. It can dispense on average $10\,mg \pm 5\%$ from any of the 288 dispensers on its carousel, into a sealed 24/96-well deep-well microtitre plate. The powders are kept in a sealed environment and are differentiated for dispensing purposes by their dispensing parameters, which are stored by in a Microsoft Access database. The database entry is pointed to by a barcode label on the dispenser—hence, adaptive dispensing.

The user interface for this multiprocessor system consists of 250 000 lines of Multithreaded/Multitasking Visual C++ code. An Access database holds dispensing parameters' information for each powder. A Dynamic Query Screen has been designed to make it easy for a chemist to view the database selectively.

Such a system can find other uses such as distributing compounds in the compound distribution centre, dispensing lyophalized microorganisms, dispensing resins for combinatorial chemistry, and any other application when powder is to be delivered precisely in a controlled environment.

### Automated system for compound library screening by MTS cell-viability assays

*Randall Engler, Kendro Lab Products, Newtown, CT, USA*
*Co-authors: C. Elliot and R. Moody*

Cell proliferation, cytotoxicity and other viability assays play an important role the drug-discovery process. Many screening programmes target anticancer compounds, requiring *in-vitro* characterization of efficacy. Additionally, all potential therapeutic compounds must be characterized for their cytotoxicity regardless of the target (ADME/Tox).

Cytokinetics has developed a system to automate the process of reagent addition, incubation and reading of cell viability assays using the MTS method of Promega, Inc. The integrated system includes hardware from a variety of sources and software developed by Cytokinetics.

Control of environmental conditions including $CO_2$, temperature and humidity are important factors in accurate and reproducible cell-based assays. The system described here includes the Heraeus® Cytomat® 6000 series incubator from Kendro for the incubation of cells.

### Automation development for high-throughput phage library screening

*Randy Yen, Genentech, Inc., San Francisco, CA, USA*
*Co-authors: Sherry Yeh and Suki Hyare*

Phage display technology is a powerful tool in genomic and drug development. It allows scientists to screen quickly billions of peptides, antibodies and cellular proteins for binding to a target. It is a vital tool in studies aimed at identifying molecules that bind to a specific target and at improving particular features of pre-existing molecules. At Genentech, we automated the binding assays for the specific target to screening the phage library. By running fully automated 384-well assay on the robotic system, we screened thousands of clones in a multiple target in 1 day and finished each library in a short time.

### Comparison and contrast of different dissolution-sampling systems using the Agilent 8453 UV-VIS in a QC laboratory

*Reem Malki, Andrx Pharmaceutical, Fort Lauderdale, FL, USA*
*Co-author: Michael K. Michaels*

An integral part of an efficient QC laboratory is thorough planning. With a new facility about to open and many diverse projects in the pipeline, the proper approach to a successful implementation is to think, research, plan, budget, set up, test, comply and train.

We knew as a laboratory that we could not function with only one type of dissolution system. Our research led us to two automated options. The first, a peristaltic pump sampling system using a single-flow cell approach, offers the detection of the sample. The second, a syringe pump sampling system using a multiflow cell approach, provided us the options to detect, detect and collect, and collect and dilute the sample. Each of these dissolution set-ups is paired with an HP8453 UV-VIS while remaining 21 CFR Part 11 compliant.

### Cross-functional team approach to implementing and managing laboratory automation in the quality control laboratory

*Timothy Reilly, Novartis Pharmaceutical Corporation, Suffern, NY, USA*

Poorly defined project goals and an inadequate infrastructure have hindered previous attempts to initiate automation projects in the Novartis Quality Control laboratory. This haphazard approach has been replaced with well-defined project expectations and agreement among the functional groups that make up the project infrastructure to support aggressively the automation project goals.

### New concept for automation of dissolution tests for biowaiver studies

*Rolf Rolli, Sotax Ltd, Allschwil, Switzerland*

New FDA regulations allow biowaivers for Class I drugs based on solubility, permeability and dissolution testing. BCS-based biowaivers can be requested for significant post-approval changes (e.g. Level 3 changes in components and compositions) to a rapidly dissolving immediate release product containing a highly soluble, highly permeable drug substance, provided that dissolution remains rapid for the post-change product and both pre-/post-change products exhibit similar dissolution profiles. Dissolution tests have to be performed at pHs 1, 4.5 and 6.8. With this regulation, automation of dissolution testing gains further importance with respect to pH changes.

A fully automated test system with the necessary software is described. This solution offers one the capability to run up to 15 USP 2 tests in series. With such a system, all steps are fully automated, from medium preparation, to tablet input and up to the printout of the reports. The system includes a very efficient cleaning device that prevents any carry-over between tests. Tests requiring baskets are handled with the Basket-Station. This system allows up to 10 USP 1 tests to be analysed. With this automated dissolution concept, biowaiver studies are executed rapidly allowing for accelerated drug development and SUPAC changes.

**Integration of reagent management and computationally biased combinatorial synthesis**

*Scott M. Harris, DuPont Pharmaceuticals Research Laboratories, San Diego, CA, USA*

One of the key aspects to successful high-throughput drug discovery is the ability to integrate several functions. These include medicinal chemistry, computational design, high-throughput synthesis and purification. A potential bottleneck in this process is a lack of established chemistries linked with readily available commercial and proprietary building blocks. Early in the process, it is critical that a protocol exists that allows for planning and synthesizing libraries, developing initial structure–activity relationships and validating screening leads.

It is also important to have access to novel building blocks and reagents for preparing targeted or focused libraries. DuPont Pharmaceuticals has developed a simple custom Web-based monomer request system that uses a JAVA GUI that allows for tracking of orders and report generation. The data are housed in a Reagent Inventory Tracking System using Oracle and the physical reagents are stored in a controlled ventilated cabinet system. The typical throughput and average turn around time will be discussed. The overall benefits of this programme are lower costs, better inventory control and an increase in efficiency in library production and chemistry.

**Automation of chemistry in a core DNA-sequencing facility**

*Shawn Hallowell, Pfizer Global R&D, Groton, CT, USA*
*Co-authors: Suzanne P. Williams, Yevette C. Clancy, Melissa T. Cronan, Shawn E. Hallowell, Michael Polchaninoff, Lance Ryley and Janice L. Palmer*

Conversion of our core DNA sequencing laboratory to the 3700 DNA analyser (AB) and sequencer for BioLIMS sequence analysis and database software (AB, genecodes) has greatly reduced the amount of manual intervention required for generating, analysing and maintaining DNA sequence data.

The set-up of the DNA sequencing reaction is the next process that has been selected for improvement. Currently, requests for sequencing from the Therapeutic Area laboratories are (1) submitted online, (2) the sample information is transferred to the 3700 plate record and (3) the addition of DNA and primers to the thermal cycle plate is performed manually.

Manual chemistry requires up to 4 h of hands-on time per day depending on the number of samples. While a liquid handler working off a file can perform the hit-picking required for core terminator chemistry, the set-up of the source racks is time-consuming and a possible source of errors. The poster will present the use of Web submission, individually bar-coded sample tubes and a liquid handler for automating chemistry set-up in a core DNA sequencing facility.

**Micro-array process industrialization**

*Shane Weber, Millennium Pharmaceuticals, Cambridge, MA, USA*

Millennium's nylon cDNA micro-array platform is successfully filling our drug- and marker-discovery platforms. This platform is effective because of its sensitivity, gene content, throughput efficiency and cost. The platform's effectiveness results from the industrialized process flow of liquid handling, printing and information tracking. Effectiveness is constantly assessed by measurements of quality and efficiency. The process flow and quality assessment will be reviewed.

**Mass-directed purification of combichem libraries**

*William R. Hall, GlaxoSmithKline, Research Triangle Park, NC, USA*
*Co-authors: Melissa Lindsay and Dean Phelps*

The Library Purification Group at Research Triangle Park is responsible for purification of both large and small libraries from discovery and targeted research. We use mass-directed preparative HPLC to purify library samples with masses of 5–100 mg per well. Our system incorporates at-column dilution, separate waste collection for each sample and UV verification of peak collection. Efficient evaporation of solvents, robotic liquid handling and reformatting, and fast QC on purified samples allows for high-throughput of purified libraries.

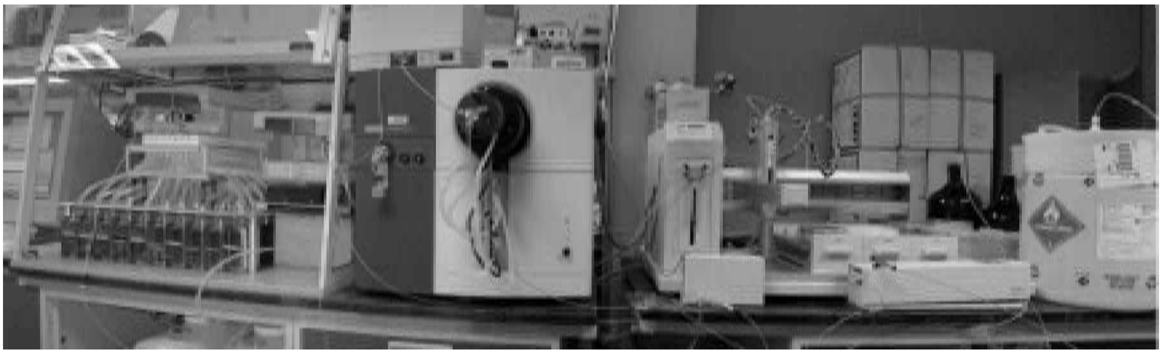

### Establishment and management of the Bristol-Myers Squibb Automated Screening Core

*Steven F. Innaimo, Bristol Myers Squibb PRI, Wallingford, CT, USA*

As part of the evolution and industrialization of the BMS Lead Discovery organization, a core group of specialized scientists focused solely on automated screening was assembled. The mission of the Automated Screening Core is to adapt and prosecute high-throughput screen campaigns on fully automated robotic screening systems.

This talk will cover the implementation of the BMS automated screening infrastructure, the rationale for the establishment of the Automated Screening Core and the organizational impact of such a group.

### Determining feasibility and parameter values for compound orders based on transfer amounts, liquid-handler capabilities and container-volume capacities

*Steven Hoffman, Bristol-Myers Squibb PRI, Princeton, NJ, USA*
*Co-author: Mark F. Russo*

An algorithm is described for determining the feasibility and calculating parameter values for one or more compound processing orders. Feasibility is determined primarily by applying a series of inequality constraints that result in a valid target concentration range within which orders can be processed. These inequality constraints are obtained from initial compound amounts, source and destination container-volume capacities, the liquid-handler transfer volume range and the amount of compound to be processed. If feasible, calculated parameter values include the dilution, transfer and top-off volumes necessary to fulfil the order. Sample code implementing the algorithm is given in Microsoft Visual Basic®.

### High-throughput screen to detect inhibitors of the 3′-5′-HCMV exonuclease

*Steven J. Conrad, Pharmacia Corporation, Kalamazoo, MI, USA*
*Co-authors: Thomas W. Pitts, Nancee Oien, Robert A. Anstadt, Roger A. Poorman, Peter A. Wells, Michael W. Wathen and Yoshihiko Yagi*

We carried out a fluorescence polarization-based (FP) high-throughput screen (HTS) to detect inhibitors of the 3′-5′-exonuclease activity of the human cytomegalovirus DNA polymerase (the HCMV exonuclease). We used a 23-mer ssDNA substrate labelled with TAMRA at the 3′ end. The emitted light from TAMRA in an intact substrate molecule was highly polarized (about 240 mP), but, upon substrate digestion with the HCMV exonuclease, the emission from TAMRA free in solution exhibited a lower polarization (about 40 mP).

The assay was designed to run in a 384-well format with a final volume of 50 ml. The $K_m$ of the HCMV exonuclease for the substrate was determined to be 20 nM. In the assay, 50 nM substrate was digested by 0.01 × stock HCMV exonuclease in 50 mM HEPES, pH 7.6, which produced a 50% reduction in mP in 8.3 min. We screened 117 843 compounds at 10 mM (2.5% DMSO). The screen identified 647 compounds that inhibited the HCMV exonuclease$^3$ 30%.

### High-throughput protein function screening for immune system target validation

*Stewart D. Chipman, Immunex, Corporation, Seattle, WA, USA*

Validation of the biochemical function of a novel gene/protein in the pathophysiology of a disease is the rate-limiting step in novel target validation for drug discovery. Many groups have applied the technology and methodology developed for small molecule high-throughput screening and clinical testing to assays that test for novel protein function. This presentation will address how we have integrated gene discovery and expression analysis, protein production and inventory capability, cell-based protein function assays, laboratory automation tools and data management systems into an integrated target discovery and validation platform for immune system targets. Topics to be discussed are automated and multiplexed cytokine detection, cognate ligand detection, calcium mobilization, high-throughput optical imaging, large-scale cell preparation, large-scale protein expression and purification, workstation style laboratory automation and automated data handling.

### Laboratory automation: a cost-effective solution to increasing laboratory workloads

*Stephen Green, Forest Laboratories Ireland Ltd, Dublin, Ireland*

Forest Laboratories, Inc., markets Celexa, an SSRI antidepressant, in the USA. The bulk tablets are produced at the manufacturing site in Dublin, Ireland. Market projections in 1999 indicated a 250% increase in volumes to be produced at the Dublin site. Several laboratory strategies to cope with this increased production were assessed and the use of automated robotic systems was shown to be the most cost-effective solution.

The introduction of the automation into the laboratory had several phases. Initially, there were physical changes required to the laboratory layout to accommodate the robots. Second, there was the qualification of the robotic systems and the training of the initial users. Finally, there was the development and validation of several analytical methods, which was performed in conjunction with the Zymark MTOV group.

### Characterization and effective use of the molecular properties of reaction vessel surfaces in high-throughput assay development

*Sven Erik Rasmussen, Nalge Nunc International, Naperville, IL, USA*
*Co-author: Nalge Nunc*

An understanding of the molecular surface of a reaction vessel is critical to achieving the sensitivity and specificity for high-throughput or high-content molecular and cell-based assays. We developed and characterized several molecular surfaces for reaction vessels that can be effectively used in genomic and proteomic assays. These surfaces, in conjunction with the physical properties of the substrate material (optical or thermal properties, for example) along with format (MicroWell, slide, or chip), are compared with regard to assay performance.

Through XPS analysis (also known as ESCA), the molecular and atomic compositions of various MicroWell surfaces have been defined. These data will be used to demonstrate the specific utilization of the surface in molecular and cell-based assays. For example, a hydrophilic, highly charged surface, MultiSorp, preferentially binds complex phospholipid molecules while excluding other molecules, including glycoproteins such as IgG. Another 'high-binding' hydrophilic surface, MaxiSorp, binds a significant amount of glycoproteins such as immunoglobins. These surfaces and others such as the Nunclon Delta cell culture surface and non-treated sterile polystyrene surfaces are molecularly defined by ESCA techniques accompanied by specific procedural examples.

Well geometry, round, square, columnar or 'shallow', influences assay performance, particularly when an active surface is used. Additional parameters come into effect when well volumes are miniaturized as in a 1536-well plate. The effect of a molecular surface changes again as the assay is further miniaturized to a two-dimensional format as in a slide or chip and the influence of the substrate material may increase.

Also important to the development of a specific, sensitive and efficient assay are the physical characteristics of various substrate materials. With regard to optical properties, the fluorescent and reflective properties of solid black and solid white as well as black or white optical bottom plates (OBP) were compared by measuring signal-to-noise ratios, light cross talk and sensitivity. Data and practical applications will be discussed.

### New polymer substrate for DNA micro-arrays

*Svend Erik Rasmussen, Nalge Nunc International, Naperville, IL, USA*
*Co-authors: T. Kristensen, K. Holmstrøm and L. Pedersen*

The quality and surface properties of the micro-array substrates are fundamental factors for a successful DNA micro-array system. At present, coated glass-slides (amine-, alehyde- or epoxy-coated) are the dominating substrates on the market. However, an often-encountered problem using these substrates is a non-homogenous surface, which can lead to problems with spot uniformity and morphology. Nunc A/S has developed a pre-activated, but uncoated, NucleoLink$^{TM}$ polymer MicroArray Slide that can be used, without any further modification, for creating DNA micro-arrays. The NucleoLink$^{TM}$ polymer MicroArray Slide can be used for both covalent attachment of DNA as well as immobilization of DNA by electrostatic forces and/or ionic bonds.

Studies of autofluorescence properties, DNA-binding capacity and DNA-hybridization efficiency have been performed and compared with conventional glass substrates. Data from oligonucleotide arrays as well as cDNA arrays will be presented.

### Flexibility paradigm in laboratory automation management

*T. C. Ramaraj, Roche Discovery Technologies, Hoffmann-La Roche, Nutley, NJ, USA*

Considerable developments and rapid changes have taken place in high-throughput screening and compound management in the last 5 years. This is a result of a concerted effort between the pharmaceutical industry, laboratory automation companies, instrument companies and manufacturers of consumable laboratory ware. The evolution of new automation technologies has fuelled the growth and prospects for various modes of automation in virtually all phases of drug-discovery research. Here, we will try to highlight our experiences in managing a complex HTS and compound management infrastructure at Hoffmann-La Roche.

The infrastructure consists of automation platforms that have evolved over several years. All require proper care and maintenance for sustained performance. Some recently installed configurations require continued validation to achieve expected levels of performance and reliability to match ever-increasing throughput requirements. Promising new automated screening technologies require careful evaluation. Validation of these new technologies also require allocation of significant resources for meeting demanding requirements of miniaturization, speed, flexibility, cost efficiency and increased performance levels. It is no easy task to steer winning methodologies to their fullest potential and at the same time take a risk in exploring unproven avenues and technologies.

To maintain a balanced perspective among available choices requires flexibility in approach, quick implementation strategies, a diverse pool of expertise and talent that can work as a team, contribute as individuals, share the successes as well as failures, learn from the mistakes, revise the expectations and still meet corporate goals and objectives every year. The presentation will also include examples from some of our most recent integration experiences, the upgrade of existing systems, the implementation of new detection modes in existing instruments and our experience with latest 384-well pipetting systems.

### Variety of automation tools for drug metabolism

*Thomas Lloyd, DuPont Pharmaceutical Co., Newark, DE, USA*

Automation continues to be developed for new diversified applications within drug metabolism. The choice of an automated tool can vary considerably depending on the specific requirements of the application. An overview of the automated tools that have been introduced at DuPont over the last 5 years will be presented along with specific application examples. An evaluation is offered for how the different tools have proliferated throughout this function.

Automation application areas include bioanalytical sample preparation techniques including solid-phase extraction, liquid–liquid extraction, protein precipitation and online approaches for *in vivo* samples, *in vitro* sample preparation processes, sample handling, data handling and training. Application areas include many functions within drug metabolism ranging from discovery through clinical trials.

### Characterization and effective use of the molecular properties of reaction vessel surfaces to achieve optimal assay performance

*Tom Cummins, Nalge Nunc International, Rochester, NY, USA*
*Co-authors: Janne N. Knudsen, Lena B. Larsen, Svend Erik Rasmussen, Tom Cummins and Joseph Granchelli*

An understanding of the molecular surface of a reaction vessel is critical to achieving the sensitivity and specificity for high-throughput or high-content molecular- and cell-based assays. We developed and characterized several molecular surfaces for reaction vessels that can be used effectively in genomic and proteomic assays. These surfaces, in conjunction with the physical properties of the substrate material (optical or thermal properties, for example) along with the format, are compared with regard to assay performance.

Through ESCA analysis, the molecular and atomic compositions of various MicroWell® surfaces have been defined. These data will be used to demonstrate the specific use of the surface in molecular and cell-based assays. For example, a hydrophilic, highly charged surface preferentially binds complex phospholipid molecules while excluding other molecules, including glycoproteins such as IgG. Another 'high-binding' hydrophilic surface, MaxiSorp™, binds a significant amount of glycoproteins such as immunoglobins.

Well geometry, round, square, columnar or 'shallow', influences assay performance, particularly when an active surface is used. Additional parameters come into effect when well volumes are miniaturized as in a 1536-well plate.

Also important to the development of a specific, sensitive and efficient assay are the physical characteristics of various substrate materials. With regard to optical properties, the fluorescent and reflective properties of solid black and solid white as well as black or white optical bottom plates (OBP) were compared by measuring signal-to-noise ratios, light cross-talk and sensitivity. Data and practical applications will be discussed.

### Intelligent automated dissolution system! A challenge worth striving for

*Umesh V. Banakar, Pharm-Assist International, Carmel, IN, USA*
*Co-author: Manoj Bagool*

Dissolution testing requirements have been extended to all oral dosage forms over the past decade. Additionally, the dissolution regulations for pharmaceutical formulations have been tightened world-wide. Furthermore, the past decade has also witnessed an increase in popularity of modified release pharmaceuticals that inherently require extended dissolution protocols. The increased emphasis on correlating dissolution and bioavailability data points to the desirability of more frequent sampling points to validate a dissolution profile. As a result, these developments have significantly increased the requirements of equipment, personnel, data processing and validation, which translates into increased production time for oral dosage forms. Thus, automation in dissolution testing has become a necessity in order to handle these demands.

Automation in dissolution testing is not new to the pharmaceutical industry. Simultaneous to the development in pharmaceutical technology over the past decade, there have been advances in automated dissolution testing, although at a slower pace. The virtual explosion in the diverse and yet specific requirements associated with a plethora of dosage form types and their respective functions have curtailed the outright all-encompassing development in automated dissolution testing. While the advantages of automated dissolution testing are unequivocal, the limitations of automated dissolution testing systems cannot be refuted. There is a constant quest to improve an existing automated system that meets specific requirements, either a unit function of the test or to accomplish a specific expectation of a dosage form, thus limiting the universality of the automated system.

Robert Kennedy once said, 'Some people watch things happen and ask the question, why; and there are those that dream of things and ask the question, why not!' It is beyond doubt that an automated dissolution testing system that addresses the current limitations of the existing ones and yet is flexible and universal enough is the need of the day. Going beyond the minimum expectations of such an automated dissolution test into its utility during early drug development and above all in simultaneous prediction of bioavailability, can be a dream worth striving for. A vision for such an automated dissolution test system will be presented.

### Development of a radiofrequency identification (RFID) data carrier interface with an analytical balance

*Jeffrey Veitch, GlaxoSmithKline, Ware, UK*
*Co-authors: Tony Allcock and James Ormand*

The use of process instrumentation such as analytical balances within the laboratory is commonplace and the need for capturing related process measurement data are often a necessity. Quite often these data need to be exported to databases and invariably to laboratory information management systems (LIMS). Laboratories regularly use process equipment such as balances, pH meters, etc. that are not attached to a dedicated PC. At GlaxoSmithKline, we have developed a way of capturing process data (e.g. weight values) directly to radiofrequency identification (RFID) smart labels, where these labels are used as data carriers.

This evaluation has proven to be a success and is potentially the stepping stone towards a more specific use of RFID within GlaxoSmithKline.

### System integration for genomics using biorobot workstations

*Achim Wehren, QIAGEN Hilden, Germany, and Valencia, CA, USA*
*Co-authors: Fred Siegmanand Carola Schade*

Automated robotic workstations are frequently used as stand-alone systems for sample purification, reaction set-up or sample re-array in the field of genomics. However, a variety of external instruments often must be integrated to provide full automation of several sequential tasks or to increase the range of applications that can be performed.

QIAGEN® Instruments provides complete solutions to automate molecular biology and liquid-handling applications by integrating BioRobot workstations with a wide range of accessory instruments, including thermal cyclers, spectrophotometers and the BioRobot RapidPlate®, a 96-channel pipetting system with capabilities for both 96- and 384-well pipetting. These integrated systems provide rapid and fully automated processing for applications such as DNA template normalization, PCR, sequencing reaction set-up and sample transfer tasks.

BioRobot® 3000 extended-arm systems are designed to accommodate complementary instruments on both the left and right sides. Additional integration of the BioRobot Twister™ increases system storage capacity for microplates, blocks and disposable tips, and allows the integration of accessory instruments with both extended-arm and standard BioRobot workstation configurations.

BioRobot® 8000 robotic workstations can be integrated with accessory instruments using the BioRobot Twister II external arm for walkaway front-end processing, e.g. from plasmid purification to final reaction set-up. The large size of the BioRobot 8000 worktable and the high storage capacity of the Twister II robotic arm allow unattended operation over extended periods.

QIAsoft™, the BioRobot Operating System, communicates with external instrument software, controls processes performed on the BioRobot and coordinates the actions of the BioRobot and external instruments.

### Dispensing precision for the SciClone™ automated liquid-handling workstation for 96-channel pipetting

*Rudy Willebrords, Janssen Research Foundation, Beerse, Belgium*

One of the first steps in drug discovery involves identification of novel compounds that interfere with therapeutically relevant biological processes. Identification of 'lead' compounds in all therapeutic areas included in a drug-discovery programme requires labour-intensive evaluation of numerous samples in a battery of therapy-targeted biological assays. To accelerate the identification of lead compounds, JRF has developed an automated high-throughput screening (HTS) based on the unattended operation of a custom Zymark-tracked robot system. Automation of enzymatic and cellular assays was realized with this system adapted to the handling of microtitre plates. The microtitre-plate technology is the basis of our screening. All compounds within our chemical library are stored and distributed in micronic tube racks or microtitre plates for screening. An efficient in-house-developed mainframe-based laboratory information management system supported all screening activities. Our experience at JRF has shown that the preparation of test compounds and making serial dilutions have been rate-limiting steps in the overall screening process. To increase compound throughput, it was necessary both to optimize the robotized assays and automate the compound supply processes. In HTS applications, one of the primary requirements is highly accurate and precise pipetting of microlitre volumes of samples into microplates. The SciClone™ is an automated liquid-handling workstation capable of both 96- and 384-channel high-precision pipetting. For high-throughput applications, the SciClone™ instrumentation can pipette a variety of liquid solutions with a high degree of accuracy and precision between microplates (interplate variability) and tip-to-tip (intraplate variability) within a single plate. The focus of this presentation is to review the liquid-handling performance of the SciClone™ system as a multipurpose instrument for pipetting aqueous or organic solutions, and virus suspensions into 96- and 384-well microplates. The capabilities of the system and the resulting benefits for our screening activities will be described.

